# Assessing skeletal muscle mass and lean body mass: an analysis of the agreement among dual X-ray absorptiometry, anthropometry, and bioelectrical impedance

**DOI:** 10.3389/fnut.2024.1445892

**Published:** 2024-08-19

**Authors:** Nicolás Baglietto, Raquel Vaquero-Cristóbal, Mario Albaladejo-Saura, Malek Mecherques-Carini, Francisco Esparza-Ros

**Affiliations:** ^1^International Kinanthropometry Chair, UCAM Universidad Católica San Antonio de Murcia, Murcia, Spain; ^2^Health Sciences PhD Program, UCAM Universidad Catolica de Murcia, Murcia, Spain; ^3^Research Group Movement Sciences and Sport (MS&SPORT), Department of Physical Activity and Sport Sciences, Faculty of Sport Sciences, University of Murcia, San Javier, Spain; ^4^Facultad de Deporte, UCAM Universidad Católica San Antonio de Murcia, Murcia, Spain

**Keywords:** skeletal muscle mass, lean body mass, DXA, anthropometry, bioimpedance, body composition

## Abstract

**Introduction:**

Methods of body composition estimation such as dual-energy X-ray absorptiometry (DXA), anthropometry, and bioimpedance (BIA) are used for the estimation of skeletal muscle mass (SMM) and lean body mass (LBM). No previous studies have examined whether these methods generate comparable results, or whether they are valid by using DXA as the reference. The aims of the present investigation were: (a) to assess the differences between DXA, anthropometry, and BIA in the estimation of SMM and LBM, taking into consideration the impact of sex and hydration status; and (b) to examine the agreement of anthropometry and BIA as compared to DXA for the estimation of SMM and LBM.

**Methods:**

A descriptive cross-sectional design was followed with 262 healthy young adults (159 males and 103 females). LBM and SMM were assessed by anthropometry with the formulas from Lee et al. and Kulkarni et al. for LBM; and Kerr (opt a), Kerr (opt b), Lee et al., Poortmans, Matiegka, Martin et al., Drinkwater and Ross, and Heymsfield et al. for SMM; by BIA with the formula reported by the TANITA MC-780-MA software for LBM and SMM; and DXA with the formula reported by the Hologic Horizon software for LBM, and the conversion by Kim et al. for SMM.

**Results:**

Significant differences were found for both SMM and LBM in kg, and percentages between most methods and formulas for the overall sample (*p* < 0.001–0.003) and divided by sex (*p* < 0.001–0.035). Hydration status did not have a significant effect on the differences between methods and formulas (*p* = 0.058–0.870). Lin’s coefficient revealed limited agreement among the majority of formulas and methods (CCC = 0.007–0.880). The Bland–Altman analysis showed significant differences in most methods and formulas, both in the overall sample and divided by sex, when using SMM and LBM with DXA as the reference (*p* < 0.001–0.030).

**Conclusion:**

There is a lack of agreement between methods and formulas for assessing SMM and LBM. Sex was found to be a significant factor in this analysis. Furthermore, significant differences were observed between most formulas and methods as compared to DXA, except for the equations to estimate SMM with anthropometry by Poortmans.

## 1 Introduction

An accurate estimation of skeletal muscle mass (SMM) is of vital importance in both health and sports (1–3). SMM is directly related to the improvement in sports performance due to its positive influence with strength, power, and endurance (4, 5). On the other hand, the accurate estimation of SMM is crucial for the diagnosis, treatment, and prevention of diseases such as sarcopenia, or even mortality (6, 7).

However, some considerations must be taken into account in the assessment of SMM in these settings. The first is that the terms SMM and lean body mass (LBM) are sometimes used as synonyms when they are not (8). SMM refers to the total weight of all skeletal muscles in the body (9, 10) analyzing body composition from a tissue perspective and positioning it at level 4 of Wang’s classification, where tissues are estimated (11). On the other hand, LBM refers to the total weight of all non-fat components of the body (9, 10). This analysis estimates molecules, positioning it at level 2 of Wang’s classification, which focuses on the molecular composition of the body (11). Specifically, it sums the molecules that constitute the non-fat components, explicitly excluding nonessential lipids. This includes water (the most predominant molecule, usually greater than 70% of the total), proteins, minerals, and other molecular components that make up LBM (11).

Secondly, several methods are used to estimate SMM and LBM, each with its specific characteristics and applications. Among these, the most commonly used in the clinical and sports fields are dual-energy X-ray absorptiometry (DXA), bioelectrical impedance (BIA), and anthropometry (12–14). DXA evaluates bone mineral content, fat, and lean soft mass by means of X-ray absorption of different tissues, and is considered by the scientific community as the reference method for analyzing bone mineral content (12, 15, 16). For estimating LBM, DXA operates directly at level 2 (molecular) dividing the body is fractionated into two components, evaluating fat mass and LBM (11), but also uses equations to indirectly estimate tissues, such as Kim’s equation (17), for the estimation of SMM at level 4 set by Wang (11). However, while this method is highly reliable and accurate, it assumes that the degree of hydration of the SMM is constant, which could be a problem in estimating the SMM and LBM component (12, 18, 19).

On the other hand, BIA is used to evaluate bioelectrical properties by analyzing the body’s resistance to the passage of an electric current, based on the premise that the conductivity of different tissues varies according to the amount of water present in them (20). It can assess molecules (level 2) through regression equations, respectively estimating fat and LBM (11, 21). Additionally, BIA can operate at level 4 when using advanced formulas to estimate tissues, allowing for the estimation of SMM, among others (11, 21). This method, although quick and easy to apply, can be unreliable due to the large number of external factors that could affect hydration status (12, 22). However, there has been little analysis of its applicability for estimating SMM and LBM (23).

Finally, anthropometry is based on taking measurements of the human body, such as skinfolds, girths, and breadths, among others (24).Through the use of equations, this tool enables the indirect estimation of SMM and LBM. For instance, sometimes anthropometry functions at level 2 (molecular), estimating LBM, and other times uses equations to estimate skeletal muscle tissue (25). However, it is important to note that it can generate problems in the interpretation of results due to possible discrepancies between the formulas used to estimate body composition by anthropometry (24, 25).

Because of the above, some studies have compared different methods for estimating SMM and LBM. The study by Reiter et al. (26) evaluated four equations for predicting appendicular LBM using BIA versus DXA in hospitalized geriatric patients, where the BIA equation proposed by Scafoglieri was highlighted as the best alternative for classifying LBM as compared to DXA (26). Another study compared SMM in climbers using DXA and the Kerr anthropometry equation, finding an overestimation of SMM using anthropometry with the Kerr formula (23). In an investigation with recreational cyclists, the Heymsfield and Drinkwater anthropometric equations showed similar results to DXA (27). Another study, focusing on male professional football players, found that the Lee anthropometric equation showed the smallest difference as compared to DXA (28). Another investigation in Brazilian male physical education students also supported Lee’s equation in agreement with DXA (29). Another study that exclusively compared anthropometric equations found that the Kerr and Matiegka formulas proved to be interchangeable, with no concordance between all the other formulas (30–32).

However, these studies suffer from significant methodological limitations. Notably, none have assessed the impact of hydration status on the comparability of results, despite water being the predominant component of both SMM and LBM (11). In addition, most studies included small samples (23, 27, 32, 33); did not include a population of both sexes (23, 28, 29); did not report the sex of the participants (27, 32); used a sample with a limited age range (29); the age range of the subjects assessed was not been reported (23, 32); they did not include a wide variety of formulas (23, 32); and they included samples of athletes from a specific sport (23, 27, 28, 32) or with specific health conditions (26), which limits the extrapolation of the data. Thus, it has not yet been generally established which methods or formulas are the most suitable to assess SMM and LBM, which often leads to the choice depending on individual preferences (32).

In view of the above, it is necessary to evaluate the differences, comparability, interchangeability, and agreement of the methods and formulas in the estimation of SMM and LBM with DXA, BIA, and anthropometry. Additionally, it is essential to scrutinize the influence of sex and hydration status on the comparability of these estimates. Prior studies have often had methodological limitations and failed to conduct a thorough analysis across different measurement tools within the same population (23, 26, 32, 33). To date, no study has comprehensively analyzed this issue using the three most commonly used methods for estimating body composition (DXA, BIA, and ANT) while controlling for significant covariates such as sex and hydration status. This is crucial given the large differences in SMM and LBM present in men and women due to sexual dimorphism (34), and the impact of hydration status on the estimation of SMM and LBM (12, 18, 19, 22, 35, 36). This study aims to fill this gap by addressing these methodological limitations.

Thus, the objectives of the present investigation are: (a) to evaluate the differences between DXA, anthropometry, and BIA in the estimation of SMM and LBM, as well as to analyzed the effect of sex and hydration level on the results obtained; and (b) to examine the agreement of anthropometry and BIA in comparison with DXA for the estimation of SMM and LBM, considering the various formulas available, and to analyzed whether sex and hydration level can influence these results.

In light of the previous findings, the hypotheses are: (a) the formulas and methods are not interchangeable, this being affected by sex and hydration level; and (b) anthropometry and BIA do not have agreement to estimate SMM and LBM as compared to DXA, this being affected by sex and hydration level.

## 2 Materials and methods

### 2.1 Design

A descriptive study was carried out using a cross-sectional design following the guidelines recommended by STROBE (37). The sample was selected using non-probability sampling. The protocol for data collection was submitted for review and approval by the Institutional Ethics Committee of the Catholic University of Murcia (code: CE072103). The present research followed the guidelines established by the World Medical Association and complied with the ethical principles stated in the Declaration of Helsinki. The participants were informed about the procedure and signed a consent form prior to starting the study.

The sample size was determined using the software Rstudio 3.15.0, specifically the package (R power package [library(pwr)]) (Rstudio Inc., Boston, MA, USA.). The significance level was set at α = 0.05. The standard deviation (SD) was set based on the percentage of SMM from previous studies (SD = 1.58) (38, 39). With an error (d) in the percentage of SMM of 0.19%, the total sample needed was 262 subjects.

### 2.2 Participants

**Figure 1:**
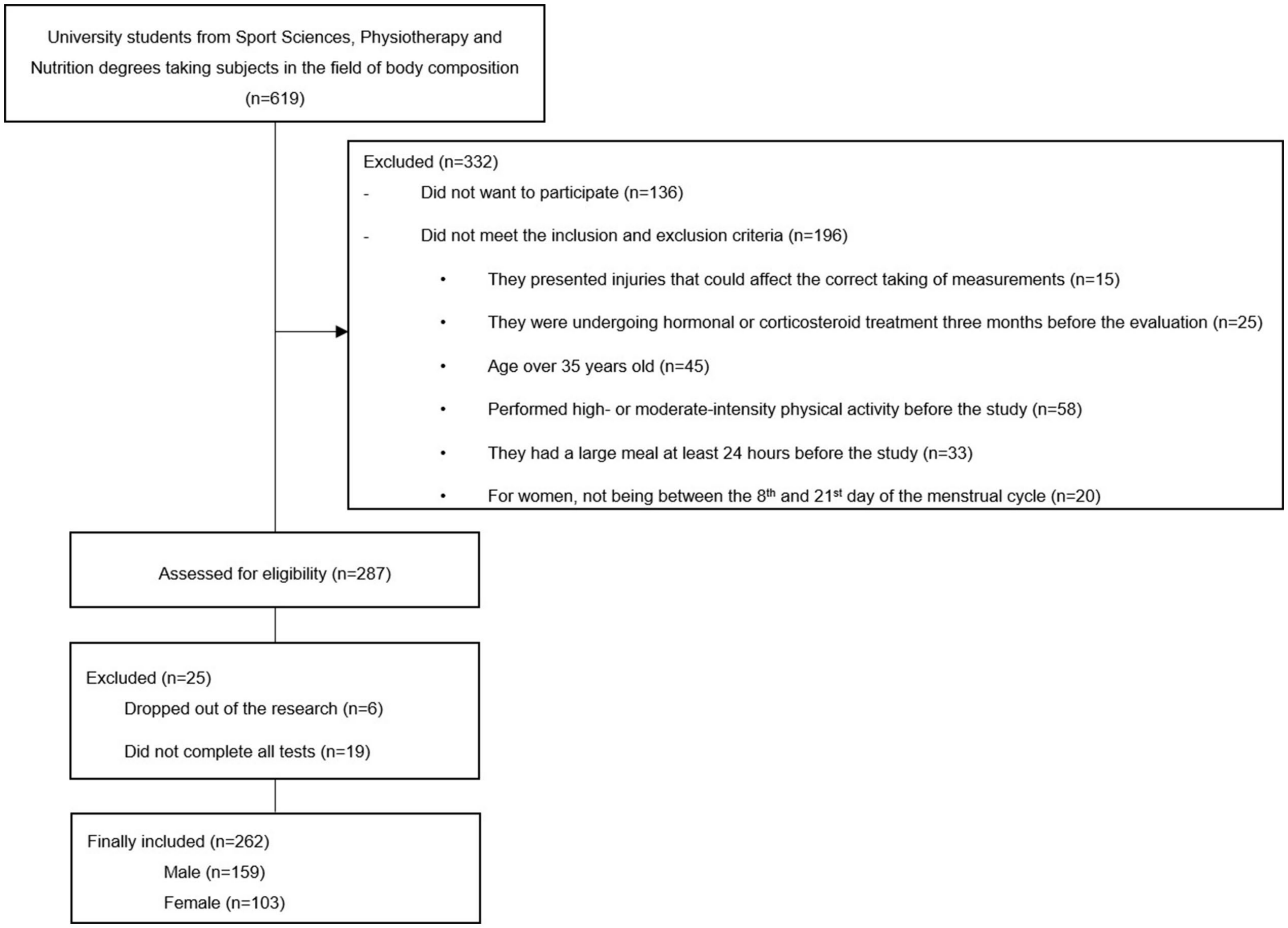
Participants’ flow chart.

A total of 262 young university students participated in the study (mean age = 23.04 ± 5.61 years-old; mean body mass = 71.58 ± 13.67 kg; mean height = 172.47 ± 9.39 cm; mean BMI = 23.92 ± 3.32 kg/m^2^). Of this group, 159 were men (mean age = 23.04 ± 5.61 years old; mean body mass = 78.35 ± 11.13 kg; mean height = 177.87 ± 6.82 cm; mean BMI = 24.74 ± 3.05 kg/m^2^) and 103 were women (mean age = 22.29 ± 5.98 years old; mean body mass = 61.13 ± 10.23 kg; mean height = 164.14 ± 6.15 cm; mean BMI = 22.65 ± 2.02 kg/m^2^). The flow diagram of the sample selection can be consulted in the Figure 1. The inclusion criteria were: (1) age between 18 and 35 years. The exclusion criteria were: (1) vigorous physical activity 24 h before the measurement, or 12 h prior in the case of moderate activity, or any type of physical exercise on the day of the measurement; (2) consumption of products with diuretic effects; (3) taking a hormonal or corticosteroid treatment 3 months before the evaluation (except for hormonal treatment to regulate the menstrual cycle); (4) for women, not being between the 8th and 21st day of the menstrual cycle; (5) taking sports supplements that could impact body composition; (6) being injured at the time of the assessment; (7) having pathologies that could affect the accumulation of SMM or LBM; and (8) failure to complete all body composition assessments.

### 2.3 Protocol

An invitation was disseminated within the virtual classroom of UCAM Universidad Católica San Antonio de Murcia (Murcia, Spain), pertaining to topics on body composition in the disciplines of Nutrition, Physiotherapy, and Sport Sciences. Students were encouraged to volunteer for a study, necessitating the completion of an initial questionnaire containing basic information. Subsequently, individuals meeting the inclusion criteria were contacted to receive additional instructions. Participants were instructed to maintain their normal intake of food and liquids in the 24 h prior to the measurements, based on previous studies (22, 40). Prior to participation, informed consent was acquired from each volunteer, and measurement appointments were arranged, taking into account the menstrual cycle for female participants.

Each volunteer was given an appointment to be assessed. All measurements were carried out in the same premises, with standardized humidity (60%) and temperature (24°C), between 9:00 and 14:00, and were conducted by the same researchers. The participants first self-completed the *ad hoc* questionnaire independently but supervised by an experienced researcher. The researcher was available to offer assistance and clarify any queries the subjects might have during this process.

Following this, a urine sample was obtained from all participants to assess their hydration levels based on urine specific gravity. Subjects were given a sterile, previously unused jar, which was clean and sealed, and asked to urinate into it.

Subsequently, DXA, anthropometry, and BIA assessments were randomly performed, by the same measurers for each test in all the measurement sessions to eliminate inter-rater technical error in the test.

#### 2.3.1 Questionnaires

An *ad hoc* questionnaire was used to collect information on socio-demographic aspects. Data were collected on sex, ethnicity, date of birth, possible pathologies that could influence the accumulation or distribution of SMM and LBM, injuries at the time of assessment, hormonal treatments or administration of corticosteroids, and diuretic intake. For female participants, the date of their last menstrual period and the time elapsed since then was recorded. In addition, data on dietary intake on the day prior to measurement were collected using a 24-h dietary recall, based on previous studies (41–43).

Physical activity was documented through a 48-h exercise recall, based on previously conducted studies (41). Participants were also asked about their regular use of sports supplements (44).

#### 2.3.2 Analysis of hydration status

In order to classify the hydration status of all participants, hydration status was analyzed according to urine specific gravity (USG) (mean = 1020.26 ± 8.52 Specific Gravity) with a MASTER-URC/Nα model refractometer (Atago, Japan), following the methodology of previous studies on a urine sample taken immediately before the measurements, collected in a sterile, previously unused, clean and sealed container (45). Hydration status was categorized based on USG values: well-hydrated (USG: ≤1.020), moderately dehydrated (USG: 1.021–1.024), and significantly dehydrated (USG: >1.024). This classification is grounded in established thresholds for urinary density (46).

#### 2.3.3 Dual-energy X-ray absorptiometry (DXA)

Each participant underwent a measurement of LBM by DXA. For the evaluation, the Hologic Horizon model scanner (Hologic Inc., USA) was used. The assessment was carried out by an experienced technician. To ensure the uniformity of the measurements, all participants were provided with specific sports clothing, and the protocols described in previous research were followed (47). Prior to the assessment, any metal objects were removed from the subject. In addition, participants were asked to urinate within 30 min prior to the measurements to minimize potential confounding variables (48). During the scan, subjects were positioned with their hands placed sideways and both feet in an internal alignment of 15° (47).

The results obtained were analyzed by means of the software Hologic APEX 13.6.0.5:5 (Hologic Inc., USA). The LBM values in kilograms (kg) and percentage reported by the system were recorded. In addition, the conversion of the results reported by the software to SMM was performed using the equation proposed by Kim et al. (17). The strategies used in the estimation of LBM and SMM by DXA can be observed in Table 1. Previous studies have reported high intra-day reliability for DXA measurements of LBM, with an intraclass correlation coefficient (ICC) of 0.994 and a standard error of measurement (SEM) of 0.42 kg, and for fat mass with an ICC of 0.998 and a SEM of 0.30 kg (49).

**Table 1:** Equations for the estimation of skeletal muscle mass and lean body mass included in the study.

	Lean body mass		Skeletal muscle mass	
DXA	Hologic Horizon software	Calculated using Hologic Horizon software (formula not available to users)	Kim et al.	Muscle mass (kg) = (1.13 * (Right leg lean mass in grams – Right leg bone mineral content in grams + Left leg lean mass in grams – Left leg bone mineral content in grams + Right arm lean mass in grams – Right arm bone mineral content in grams + Left arm lean mass in grams – Left arm bone mineral content in grams)/1,000) – (0.02 * Age in years) + (0.61 * (1 if male; 0 if female)) + 0.97
BIA	TANITA MC-780-MA software	Calculated using TANITA MC-780-MA software	TANITA MC-780-MA software	Calculated using TANITA MC-780-MA software (formula not available to users)
ANT	Lee et al.	Males: Lean body mass (kg) = –1.401 – (0.01 * Age in years) + (0.1 * Height in cm) + (0.632 * Body mass in kg) – (0.225 * Waist girth in cm) + (0.315 * Relaxed arm girth in cm) + (0.091 * Calf girth in cm) + (0.04 * Thigh girth in cm) – (0.304 * Triceps skinfold in cm) – (0.021 * Subscapular skinfold in cm) + 0.097 Females: Lean body mass (kg) = –9.193 – (0.045 * Age in years) + (0.158 * Height in cm) + (0.41 * Body mass in kg) – (0.04 * Waist girth in cm) + (0.095 * Relaxed arm girth in cm) + (0.193 * Calf girth in cm) – (0.105 * Thigh girth in cm) – (0.152 * Triceps skinfold in cm) – (0.004 * Subscapular skinfold in cm) + 0.082	Kerr (opt. 1)	Muscle mass (kg) = [(Z-score muscle * 4.4) + 24.5]/(170.18/Height in cm)^3^; where: *Z*-score muscle = [(Sum of corrected girths in cm * (170.18/Height in cm) − 207.21)/13.74]; and Sum of corrected girths in cm = (Relaxed arm girth in cm-π * Triceps skinfold in cm) + Forearm girth in cm + (Chest girth in cm-π * Subscapular skinfold in cm) + (1 cm Gluteal Thigh Girth in cm-π * Thigh skinfold in cm) + (Calf girth in cm-π * Calf skinfold in cm)
	Kulkarni et al.	Males: Lean body mass (kg) = 10.385 – (0.005 * Age in years) + (0.103 * Height in cm) + (0.68 * Body mass in kg) + (0.288 * Relaxed arm girth in cm) + (0.13 * Calf girth in cm) – (0.183 * Hip girth in cm) – (5.278 * log(Suprailiac skinfold in cm + Subscapular skinfold in cm + Triceps skinfold in cm + Biceps skinfold in cm)) Females: Lean body mass (kg) = 10.632 – (0.009 * Age in years) + (0.102 * Height in cm) + (0.592 * Body mass in kg) + (0.055 * Relaxed arm girth in cm) + (0.043 * Calf girth in cm) – (0.158 * Hip girth in cm) – (3.174 * log(Suprailiac skinfold in cm + Subscapular skinfold in cm + Triceps skinfold in cm + Biceps skinfold in cm))	Kerr (opt. 2)	Muscle mass (kg) = [(*Z*-score muscle * 4.4) + 24.5]/(170.18/Height in cm)^3^; where: *Z*-score muscle = [(Sum of corrected girths in cm * (170.18/Height in cm) − 207.21)/13.74]; and Sum of corrected girths in cm = (Relaxed arm girth in cm-π * Triceps skinfold in cm) + Forearm girth in cm + (Chest girth in cm-π * Subscapular skinfold in cm) + (Thigh middle girth in cm-π * Thigh skinfold in cm) + (Calf girth in cm-π * Calf skinfold in cm)
			Lee et al.	Muscle mass (kg) = Height in m * [0.00744 * (Relaxed arm girth in cm-π * Triceps skinfold in cm)^2^ + 0.00088 * (Thigh middle girth in cm-π * Thigh skinfold in cm)^2^ + 0.00441 * (Calf girth in cm-π * Calf skinfold in cm)^2^] + 2.4 * (1 if male; 0 if female) − 0.048 * Age in years + (1 if black race; 0 if Caucasian race; and − 1 if Asian race) + 7.8
			Poortmans	Muscle mass (kg) = Height in m * [0.0064 * (Relaxed arm girth in cm-π * Triceps skinfold in cm)^2^ + 0.0032 * (Thigh middle girth in cm-π * Thigh skinfold in cm)^2^ + 0.0015 * (Calf girth in cm-π * Calf skinfold in cm)^2^] + 2.56 * (1 if male; 0 if female) + 0.136 * Age in years
			Matiegka	Muscle mass (kg) = Height in m * [[(Arm relaxed girth in cm/π-Triceps skinfold in cm) + (Thigh middle girth in cm-π * Thigh skinfold in cm) + (Calf girth in cm-π * Calf skinfold in cm) + (Chest girth in cm-π * Subscapular skinfold in cm)] ^0.125^]^2^ * 6.41
			Martin et al.	Muscle mass (kg) = [(Height in cm * (0.0553 * (Mid-Thigh Girth in cm - π * Thigh Skinfold in cm)^2^ + 0.0987 * (Forearm Girth in cm)^2^ + 0.0331 * (Calf Girth in cm – π * Calf Skinfold in cm)^2^) – 2445) * 0.001]
			Drinkwater and Ross	Muscle mass (kg) = [(Z-score Drinkwater and Ross * 2.99 + 25.55)/(170.18/Height in cm)^3^]; where: *Z*-score Drinkwater and Ross = [(Sum of corrected girths in cm * (170.18/Height in cm) – 182.07)/3.09]; and: Sum of corrected girths in cm = [(Relaxed arm girth in cm – π * Triceps skinfold in cm) * (170.18/Height in cm) – 22.05]/1.91 + [(Chest girth in cm – π * Subscapular skinfold in cm) * (170.18/Height in cm) – 82.46]/4.86 + [(Thigh middle girth in cm – π * Thigh skinfold in cm) * (170.18/Height in cm) – 47.34]/3.59 + [(Calf girth in cm – π * Calf skinfold in cm) * (170.18/Height in cm) – 30.22]/1.97 + Forearm girth in cm.
			Heymsfield et al.	Muscle mass (kg) = Height in cm * (0.0264 + (0.0029 * Braquial Muscle Area)); where: Braquial Muscle Area (cm^2^) = [(Relaxed arm girth in cm - (Triceps skinfold in cm) * 3.415927)^2^]/(4 * π);

* DXA, dual-energy X-ray Absorptiometry; BIA, bioimpedance; ANT: anthropometry.

#### 2.3.4 Bioimpedance analysis (BIA)

Each participant was measured with BIA using the TANITA MC-780-MA model (Tanita Cooperation, Japan). This device consists of a segmental multi-frequency device, which uses specific measurement frequencies (5 kHz/50 kHz/250 kHz) through a configuration composed of eight electrodes (50). Throughout the evaluation process, the guidelines established by both the manufacturer and previous research were strictly followed, including urination within 30 min prior to measurements, and questions were also asked about water consumption or dietary factors that might affect total body water levels in order to minimize potential sources of variability (40). Each participant was evaluated in tight-fitting sports clothing, making sure to remove all metallic elements before the evaluation. Prior to and during the analysis, the arms of the subjects were positioned at 30° with respect to the trunk of the body, and the legs at 45°. Before carrying out the evaluation, the location of the electrodes in direct contact with the individual’s body was carefully verified (51). After the measurement was taken, the TANITA MC-780-MA system software (Tanita Cooperation, Japan) was used to obtain the percentage and kilograms of SMM and LBM (Table 1). Previous studies have reported high intra-day reliability for BIA measurements of LBM, with an intraclass correlation coefficient (ICC) of 0.987 and a standard error of measurement (SEM) of 0.53 kg, and for fat mass, with an ICC of 0.990 and a SEM of 0.40 kg (49).

#### 2.3.5 Anthropometry

The evaluation of the anthropometric variables was carried out following the protocols established by the International Society for the Advancement in Kinanthropometry (ISAK) (52). Two basic measurements (body mass and stretch stature), four skinfolds (triceps, subscapular, thigh and calf); and eight girths (arm relaxed, forearm, chest, waist, hips, thigh 1 cm gluteal, thigh middle and calf) were assessed, following the guidelines from the ISAK (52). To measure body mass, a TANITA MC-780-MA scale (Tanita Cooperation, Japan) with an accuracy of 0.1 kg was used, while height was measured using a SECA 213 stadiometer (SECA, Germany) with an accuracy of 0.1 cm. Skinfolds were assessed using a Harpenden caliper (Harpenden, London, UK), with an accuracy of 0.2 mm. Girths were measured using an inextensible millimeter Lufkin W606PM tape (Lufkin, USA) with an accuracy of 0.1 cm. All instruments used were pre-calibrated to ensure accuracy and minimize the margin of error in the measurements.

These measurements were performed by an anthropometrist with an up-to-date level 3 certification issued by the ISAK. Each measurement was performed in duplicate, and in case of a difference of more than 5% for the skinfolds and 1% for the rest of the variables between measurements, a third evaluation was carried out. The final value used for data analysis was the mean when two measurements were taken, or the median for variables where three measurements were taken. Furthermore, intra-evaluator accuracy was assessed by calculating the technical error of measurement (TEM). The intra-evaluator TEM values were 0.01% for basic measurements, 0.7% for skinfolds, and 0.3% for girths. Anthropometric measurements of percentage fat mass has been reported to have an ICC of 0.991 and a SEM of 0.63% in previous studies (53).

After the assessments, LBM and SMM values in kg and percentage were calculated according to different equations. The equations for estimating LBM were those by Lee et al. (54), and Kulkarni et al. (55). For SMM, the formulas proposed by Kerr were used, using both proposed for its calculation, including the thigh 1 cm gluteal girth (Kerr – opt 1), as well as with the thigh middle girth (Kerr – opt 2) (30), Matiegka (31), Kulkarni (55), Lee et al. (56), Poortmans (57), Martin et al. (58), Drinkwater and Ross (59), and Heymsfield et al. (60). The equations used in the estimation of LBM and SMM by anthropometry can be found in Table 1.

#### 2.3.6 Rationale for formula selection

The formulas used for estimating SMM and LBM by DXA and BIA were those applied by the software of the devices used.

The Kim 2002 equation was selected for the estimation of SMM via DXA due to its widespread use and acceptance in the scientific community. This particular equation is recognized for its reliability and validity across diverse populations. Its frequent application in numerous studies underscores its robustness and credibility (17, 23, 27, 28, 57, 61).

Regarding the formulas used by anthropometry, were selected based on their widespread use and compatibility with the variables measured through the ISAK protocol (6, 30, 31, 55, 56, 62, 63). The chosen formulas, such as those by Lee et al., Kerr, Poortmans, Matiegka, Martin, Drinkwater and Ross, Heymsfield et al., and Kulkarni, are well-established in the literature and have been utilized in previous studies (23, 25, 27–29, 32, 64). These formulas are aligned with the demographic and physical characteristics of our sample (6, 30, 31, 55, 56, 62, 63); Furthermore, current study included all recommended equations from the consensus for anthropometric assessment ensures comprehensive evaluation (65).

### 2.4 Statistical analysis

The normal distribution with the Kolmogorov–Smirnov test, kurtosis, and asymmetry of the variables were calculated. Levene’s test was used to assess the homogeneity of the variables. The analysis of skewness and kurtosis showed a platykurtic distribution for all variables. As a normal and homogeneous distribution of the variables was found, parametric tests were performed. Descriptive statistics were carried out for all the variables analyzed. Sex differences in USG were analyzed with a Student’s t-test. Differences between the LBM and SMM results, and the methods used, were subjected to a one-way analysis of covariance (ANCOVA) for repeated measurements, including the different formulas for estimating LBM and SMM, both in kg and in percentage. The influence of the covariates “sex” and “hydration status” was also assessed. A Bonferroni *post hoc* adjustment was applied to explore pairwise differences between equations and methods. The effect size for pairwise comparisons was quantified through the partial Eta-squared coefficient (η^2^_p_). Differences were also accompanied by the inclusion of confidence intervals (CI) at a 95% confidence level. The software used in the statistical analysis was SPSS (v.23, IBM, Endicott, NY, USA). The agreement between equations and methods was determined using Lin’s concordance correlation coefficient (CCC), including precision (ρ) and accuracy (Cb) indexes, as well as with McBride’s strength concordance (almost perfect >0.99; substantial >0.95 to 0.99; moderate = 0.90–0.95; and poor <0.90), following previous research (66).The Bland–Altman test was used to determine the agreement of different anthropometry and BIA equations with respect to DXA values. The regression equation for the model was also calculated. The software used to perform the Bland–Altman test and Lin’s CCC was MedCalc Statistical Software v.20.106 (Mariakerke, Belgium). For all the statistical tests, the significance level was set *a priori* to *p* ≤ 0.05.

## 3 Results

The mean USG value of the general sample was 1020.26 ± 8.52, with a minimum value of 1,002 and a maximum value of 1,036; with no significant differences according to sex (*t* = 2.042; *p* = 0.052; ICC = –0.078; 4.297).

Table 2 shows the outcomes derived from the descriptive statistical examination conducted on the general population, as well as the values for the men and women groups. The data shown encompasses key metrics such as means, standard deviations, minimum, and maximum values.

**Table 2:** Descriptive analysis of kg and percentages of skeletal muscle mass and lean body mass in the general sample and divided into males and females.

				General sample (*n* = 262)		Male sample (*n* = 159)		Female sample (*n* = 103)	
Variable	Method	Formula	Unit	Mean ± SD	Min.-Max.	Mean ± SD	Min.-Max.	Mean ± SD	Min.-Max.
SMM	DXA	Kim et al.	Kg	26.77 ± 7.33	12.38;43.59	31.66 ± 4.82	19.79;43.59	19.23 ± 2.62	12.38;25.67
	BIA	TANITA MC-780-MA soft.		32.17 ± 6.54	19.10;48.60	36.43 ± 4.18	26.70;48.60	25.60 ± 3.25	19.10;36.30
	ANT	Kerr (opt. 1)		30.95 ± 8.14	16.71;51.91	36.06 ± 5.72	22.07;51.91	23.07 ± 3.93	16.71;36.32
		Kerr (opt. 2)		28.73 ± 8.19	14.41;48.23	33.97 ± 5.60	19.19;48.23	20.65 ± 3.82	14.41;33.69
		Lee et al.		28.38 ± 7.08	15.89;44.77	33.30 ± 41.13	23.92;44.77	20.79 ± 2.38	15.89;28.13
		Poortmans		27.14 ± 7.99	13.12;45.39	32.53 ± 5.04	20.14;45.39	18.81 ± 2.92	13.12;28.32
		Matiegka		30.92 ± 2.34	25.50;37.33	32.41 ± 1.52	28.54;37.33	28.61 ± 1.28	25.50;32.15
		Martin et al.		36.62 ± 9.99	17.94;62.33	42.90 ± 7.03	27.37;62.33	26.94 ± 4.77	17.94;43.48
		Drinkwater y Ross		29.56 ± 7.12	16.94;47.57	34.24 ± 4.69	23.92;47.57	22.36 ± 3.03	16.94;31.47
		Heymsfield et al.		30.73 ± 10.91	13.73;57.44	37.74 ± 7.93	23.09;57.44	19.92 ± 3.52	13.73;32.40
SMM	DXA	Kim et al.	%	37.15 ± 6.15	22.34;51.57	40.60 ± 4.67	25.98;51.57	31.81 ± 3.95	22.34;41.22
	BIA	TANITA MC-780-MA soft.		44.98 ± 3.98	33.46;56.28	46.74 ± 2.97	36.32;54.82	42.27 ± 3.81	33.46;56.28
	ANT	Kerr (opt. 1)		42.80 ± 5.08	30.13;54.44	46.01 ± 3.28	36.90;54.44	37.83 ± 2.90	30.13;45.32
		Kerr (opt. 2)		39.59 ± 5.80	27.21;53.16	43.33 ± 3.63	32.08;53.16	33.82 ± 3.21	27.21;42.75
		Lee et al.		39.49 ± 5.48	24.38;51.44	42.76 ± 3.67	29.80;51.44	34.45 ± 3.70	24.38;44.72
		Poortmans		37.44 ± 6.38	22.17;50.78	41.61 ± 3.82	28.09;50.78	31.02 ± 3.60	22.17;40.84
		Matiegka		44.26 ± 5.98	29.76;64.38	41.99 ± 4.65	30.66;53.64	47.77 ± 6.12	29.76;64.38
		Martin et al.		50.59 ± 6.85	32.62;67.00	54.74 ± 4.69	36.68;67.00	44.18 ± 4.19	32.62;55.25
		Drinkwater y Ross		41.08 ± 4.58	27.86;51.43	43.83 ± 3.17	33.97;51.43	36.84 ± 2.84	27.86;44.46
		Heymsfield et al.		42.12 ± 9.83	24.87;65.60	48.14 ± 7.35	32.95;65.60	32.84 ± 4.53	24.87;46.59
LBM	DXA	Hologic Horizon software	Kg	51.99 ± 12.02	30.48;82.51	59.83 ± 8.09	41.36;82.51	39.88 ± 4.93	30.48;55.18
	BIA	TANITA MC-780-MA soft.		53.94 ± 11.02	32.00;81.70	61.17 ± 7.05	44.70;81.70	42.79 ± 5.16	32.00;57.60
	ANT	Lee et al.		51.27 ± 11.61	31.28;77.16	59.26 ± 6.94	43.52;77.16	45.43 ± 4.16	31.28;54.65
		Kulkarni et al.		59.99 ± 13.57	36.03;89.50	69.42 ± 7.63	53.47;89.50	38.93 ± 5.33	36.03;66.48
LBM	DXA	Hologic Horizon software	%	73.51 ± 8.29	52.11;88.87	77.93 ± 6.44	57.75;88.87	66.69 ± 5.87	52.11;81.55
	BIA	TANITA MC-780-MA soft.		75.36 ± 6.56	56.00;92.10	78.43 ± 4.90	61.00;92.10	70.62 ± 5.94	56.00;85.60
	ANT	Lee et al.		71.37 ± 6.95	53.17;84.37	75.95 ± 3.70	63.97;84.37	64.31 ± 4.41	53.17;76.03
		Kulkarni et al.		83.47 ± 7.82	64.67;96.78	89.02 ± 3.47	79.44;96.78	74.92 ± 3.98	64.67;86.16

* SMM, skeletal muscle mass; LBM, lean body mass; DXA, Dual-energy X-ray absorptiometry; BIA, Bioimpedance analysis; ANT, anthropometry; Soft, software; Kg, kilograms; %, percentages; SG, Specific Gravity; CS, Color scale.

### 3.1 Analysis of differences

The overall analysis of all methods is presented using ANOVA, ANCOVA and Bonferroni *post hoc* tests to assess the agreement and differences between the different methods for estimating SMM and LBM for the overall and sex-divided sample.

Table 3 shows the results of the ANOVA and ANCOVA performed to analyze separately the effect of the covariates sex and hydration status in both the overall sample and the sample segmented according to sex. For both the overall sample and the differentiated sample of men and women, significant differences were found between all the methods used to estimate SMM or LBM in both kilograms and in percentages (*p* < 0.001–0.021). Sex showed a significant effect on the comparability of all the variables analyzed (*p* = 0.023- < 0.001). On the other hand, hydration status did not show a significant effect on the comparability between methods (*p* = 0.058-0.870).

**Table 3:** Analysis of differences in skeletal muscle mass and lean body mass (kg and percentage) between DXA, Anthropometry, and BIA for the total sample and according to sex.

	ANOVA			Variable*Hydration status			Variable*Sex		
	*F*	*p*	Ƞ2p	*F*	*p*	Ƞ2p	*F*	*P*	Ƞ2p
General sample (*n* = 262)									
Skeletal muscle mass (Kg)	169.091	<0.001	0.393	0.179	0.673	0.001	5.230	0.023	0.020
Skeletal muscle mass (%)	181.684	<0.001	0.410	0.027	0.870	<0.001	14.390	<0.001	0.052
Lean body mass (Kg)	32.975	<0.001	0.112	3.635	0.058	0.014	25.786	<0.001	0.090
Lean body mass (%)	5.371	0.021	0.020	2.998	0.085	0.011	17.230	<0.001	0.062
Males (*n* = 159)									
Skeletal muscle mass (Kg)	105.737	<0.001	0.401						
Skeletal muscle mass (%)	106.988	<0.001	0.404						
Lean body mass (Kg)	51.896	<0.001	0.247						
Lean body mass (%)	19.831	<0.001	0.112						
Females (*n* = 103)									
Skeletal muscle mass (Kg)	66.604	<0.001	0.395						
Skeletal muscle mass (%)	74.133	<0.001	0.421						
Lean body mass (Kg)	23.442	<0.001	0.166						
Lean body mass (%)	57.761	<0.001	0.362						

* Kg, kilograms; %, percentages.

Table 4 shows the Bonferroni adjustment for both the general sample and the sample divided between men and women with respect to SMM in kilograms. In the general sample, significant differences were found between all methods and formulas (*p* < 0.001-0.003); with the exception of DXA with anthropometry according to Poortmans (*p* = 1.000); and the anthropometry proposals by Kerr (opt. 1) with Matiegka and Heymsfield (*p* = 1.000), Kerr (opt. 2) with Lee (*p* = 0.253), and Matiegka with Heymsfield (*p* = 1.000). In the men’s group, all methods and formulas showed significant differences (*p* < 0.001–0.026), with the exception of DXA with anthropometry according to Matiegka (*p* = 0.545); BIA with the anthropometry equations of Kerr (opt. 1) and Heymsfield (*p* = 0.093-1.000); and the anthropometry proposals of Kerr (opt. 2) with Drinkwater (*p* = 1.000), and Matiegka with Poortmans (*p* = 1.000). Finally, in the female group, all equations presented significant differences (*p* < 0.001–0.007), with the exception of DXA with anthropometry according to Heymsfield and Poortmans (*p* = 0.213-0.792); and the anthropometry equations of Kerr (opt. 2) and Lee (*p* = 1.000).

**Table 4:** Differences between methods of estimating skeletal muscle mass in kg.

	General sample (*n* = 262)			Males (*n* = 159)			Females (*n* = 103)		
Comparison	Mean differences ± SD	95%CI (min; max)	*p-*value	Mean differences ± SD	95%CI (min; max)	*p*-value	Mean differences ± SD	95%CI (min; max)	*p-*value
DXA vs. BIA	–5.40 ± 0.14	–5.86; –4.95	<0.001	–4.78 ± 0.18	–5.37; –4.18	<0.001	–6.37 ± 0.19	–6.99; –5.75	<0.001
DXA vs. ANT Kerr (opt. 1)	–4.19 ± 0.19	–4.82; –3.56	<0.001	–4.41 ± 0.27	–5.32; –3.50	<0.001	–3.84 ± 0.24	–4.64; –3.04	<0.001
DXA vs. ANT Kerr (opt. 2)	–1.96 ± 0.18	–2.57; –1.35	<0.001	–2.31 ± 0.26	–3.18; –1.45	<0.001	–1.42 ± 0.23	–2.19; –0.65	<0.001
DXA vs. ANT Matiegka	–4.15 ± 0.33	–5.22; –3.07	<0.001	–0.75 ± 0.30	–1.74; 0.23	0.545	–9.38 ± 0.20	–10.05; –8.72	<0.001
DXA vs. ANT Martin	–9.85 ± 0.25	–10.66; –9.04	<0.001	–11.24 ± 0.32	–12.29; –10.19	<0.001	–7.72 ± 0.28	–8.66; –6.77	<0.001
DXA vs. ANT Drinkwater	–2.80 ± 0.13	–3.22; –2.38	<0.001	–2.58 ± 0.18	–3.19; –1.97	<0.001	–3.13 ± 0.16	–3.66:–2.61	<0.001
DXA vs. ANT Heymsfield	–3.96 ± 0.32	–5.02; –2.91	<0.001	–6.08 ± 0.43	–7.50; –4.66	<0.001	–0.69 ± 0.24	–1.50; 0.11	0.213
DXA vs. ANT Lee	–1.62 ± 0.13	–2.05; –1.18	<0.001	–1.65 ± 0.20	–2.32; –0.98	<0.001	–1.56 ± 0.14	–2.02; –1.11	<0.001
DXA vs. ANT Poortmans	–0.37 ± 0.16	–0.90; 0.16	1.000	–0.88 ± 0.23	–1.65; –0.10	0.011	0.42 ± 0.17	–0.16; 0.99	0.792
BIA vs. ANT Kerr (opt. 1)	1.22 ± 0.16	0.69; 1.74	<0.001	0.37 ± 0.20	–0.30; 1.03	1.000	2.53 ± 0.21	1.84; 3.23	<0.001
BIA vs. ANT Kerr (opt. 2)	3.44 ± 0.16	2.90; 3.98	<0.001	2.46 ± 0.20	1.81; 3.12	<0.001	4.96 ± 0.21	4.25 ± 5.66	<0.001
BIA vs. ANT Matiegka	1.26 ± 0.28	0.35; 2.17	<0.001	4.02 ± 0.25	3.21; 4.83	<0.001	–3.01 ± 0.24	–3.83 ± –2.19	<0.001
BIA vs. ANT Martin	–4.45 ± 0.25	–5.29; –3.61	<0.001	–6.46 ± 0.29	–7.42; –5.50	<0.001	–1.34 ± 0.25	–2.19 ± –0.50	<0.001
BIA vs. ANT Drinkwater	2.61 ± 0.09	2.30; 2.92	<0.001	2.19 ± 0.11	1.82; 2.57	<0.001	3.24 ± 0.14	2.76 ± 3.72	<0.001
BIA vs. ANT Heymsfield	1.44 ± 0.34	0.31; 2.57	0.002	–1.31 ± 0.42	–2.69; 0.08	0.093	5.68 ± 0.24	4.87 ± 6.49	<0.001
BIA vs. ANT Lee	3.79 ± 0.13	3.37; 4.21	<0.001	3.13 ± 0.15	2.62; 3.63	<0.001	4.81 ± 0.19	4.18 ± 5.44	<0.001
BIA vs. ANT Poortmans	5.04 ± 0.16	4.50; 5.57	<0.001	3.90 ± 0.19	3.28; 4.52	<0.001	6.79 ± 0.20	6.12 ± 7.46	<0.001
ANT Kerr (opt. 1) vs. ANT Kerr (opt. 2)	2.23 ± 0.05	2.07; 2.38	<0.001	2.10 ± 0.06	1.89; 2.30	<0.001	2.42 ± 0.07	2.19; 2.66	<0.001
ANT Kerr (opt. 1) vs. ANT Matiegka	0.04 ± 0.38	–1.22; 1.30	1.000	3.65 ± 0.38	2.39; 4.91	<0.001	–5.54 ± 0.31	–6.59; –4.49	<0.001
ANT Kerr (opt. 1) vs. ANT Martin	–5.67 ± 0.16	–6.20; –5.13	<0.001	–6.83 ± 0.20	–7.49; –6.17	<0.001	–3.87 ± 0.16	–4.40; 3.35	<0.001
ANT Kerr (opt. 1) vs. ANT Drinkwater	1.39 ± 0.10	1.05; 1.73	<0.001	1.83 ± 0.14	1.37; 2.29	<0.001	0.71 ± 0.13	0.28; 1.13	<0.001
ANT Kerr (opt. 1) vs. ANT Heymsfield	0.22 ± 0.26	–0.62; 1.07	1.000	–1.67 ± 0.32	–2.73; –0.62	<0.001	3.15 ± 0.22	2.43; 3.87	<0.001
ANT Kerr (opt. 1) vs. ANT Lee	2.57 ± 0.14	2.12:3.02	<0.001	2.76 ± 0.18	2.15; 3.37	<0.001	2.28 ± 0.20	1.62; 2.94	<0.001
ANT Kerr (opt. 1) vs. ANT Poortmans	3.81 ± 0.12	3.43; 4.20	<0.001	3.53 ± 0.14	3.06; 4.00	<0.001	4.26 ± 0.19	3.61; 4.90	<0.001
ANT Kerr (opt. 2) vs. ANT Matiegka	–2.19 ± 0.39	–3.46; –0.92	<0.001	1.56 ± 0.37	0.33; 2.79	0.002	–7.96 ± 0.31	–9.02; 6.91	<0.001
ANT Kerr (opt. 2) vs. ANT Martin	–7.89 ± 0.15	–8.40; –7.39	<0.001	–8.93 ± 0.19	–9.55 ± –8.30	<0.001	–6.30 ± 0.16	–6.84; –5.76	<0.001
ANT Kerr (opt. 2) vs. ANT Drinkwater	–0.84 ± 0.10	–1.18; –0.50	<0.001	–0.27 ± 0.13	–0.70; 0.16	1.000	–1.72 ± 0.13	–2.15; –1.28	<0.001
ANT Kerr (opt. 2) vs. ANT Heymsfield	–2.00 ± 0.24	–2.81; –1.20	<0.001	–3.77 ± 0.31	–4.80; –2.73	<0.001	0.73 ± 0.18	0.11; 1.35	0.007
ANT Kerr (opt. 2) vs. ANT Lee	0.35 ± 0.12	–0.06; 0.75	0.253	0.66 ± 0.16	0.12; 1.21	0.003	–0.15 ± 0.18	–0.75; 0.16	1.000
ANT Kerr (opt. 2) vs. ANT Poortmans	1.59 ± 0.10	1.27; 1.91	<0.001	1.44 ± 0.12	1.05; 1.82	<0.001	1.84 ± 0.17	1.28; 2.39	<0.001
ANT Matiegka vs. ANT Martin	–5.71 ± 0.49	–7.33; –4.09	<0.001	–10.48 ± 0.48	–12.07; –8.90	<0.001	1.67 ± 0.39	0.37; 2.97	0.002
ANT Matiegka vs. ANT Drinkwater	1.35 ± 0.31	0.33; 2.37	0.001	–1.83 ± 0.28	–2.77; –0.89	<0.001	6.25 ± 0.21	5.55; 6.95	<0.001
ANT Matiegka vs. ANT Heymsfield	0.18 ± 0.56	–1.66; 2.02	1.000	–5.33 ± 0.56	–7.20; –3.46	<0.001	8.69 ± 0.31	7.66; 9.72	<0.001
ANT Matiegka vs. ANT Lee	2.53 ± 0.31	1.50; 3.57	<0.001	–0.89 ± 0.25	–1.74; –0.05	0.026	7.82 ± 0.18	7.21; 8.43	<0.001
ANT Matiegka vs. ANT Poortmans	3.78 ± 0.37	2.55; 5.00	<0.001	–0.12 ± 0.33	–1.21; 0.96	1.000	9.80 ± 0.23	9.02; 10.58	<0.001
ANT Martin vs. ANT Drinkwater	7.06 ± 0.20	6.39; 7.73	<0.001	8.66 ± 0.24	7.88; 9.44	<0.001	4.58 ± 0.19	3.93; 5.23	<0.001
ANT Martin vs. ANT Heymsfield	5.89 ± 0.26	5.04; 6.75	<0.001	5.16 ± 0.38	3.91; 6.40	<0.001	7.02 ± 0.28	6.07; 7.97	<0.001
ANT Martin vs. ANT Lee	8.24 ± 0.22	7.50; 8.97	<0.001	9.59 ± 0.28	8.67; 10.51	<0.001	6.15 ± 0.26	5.27; 7.04	<0.001
ANT Martin vs. ANT Poortmans	9.49 ± 0.17	8.93; 10.04	<0.001	10.36 ± 0.21	9.68; 11.04	<0.001	8.13 ± 0.23	7.35; 8.91	<0.001
ANT Drinkwater vs. ANT Heymsfield	–1.16 ± 0.29	–2.12; –0.21	0.003	–3.50 ± 0.35	–4.66; –2.34	<0.001	2.44 ± 0.20	1.78; 3.10	<0.001
ANT Drinkwater vs. ANT Lee	1.18 ± 0.08	0.91; 1.45	<0.001	0.93 ± 0.11	0.58 ± 1.29	<0.001	1.57 ± 0.12	1.18; 1.97	<0.001
ANT Drinkwater vs. ANT Poortmans	2.43 ± 0.11	2.07; 2.79	<0.001	1.70 ± 0.13	1.29 ± 2.12	<0.001	3.55 ± 0.14	3.09; 4.01	<0.001
ANT Heymsfield vs. ANT Lee	2.35 ± 0.27	1.45; 3.24	<0.001	4.43 ± 0.34	3.29; 5.57	<0.001	–0.87 ± 0.18	–1.47; –0.28	<0.001
ANT Heymsfield vs. ANT Poortmans	3.59 ± 0.24	2.82; 4.37	<0.001	5.20 ± 0.31	4.16; 6.25	<0.001	1.11 ± 0.17	0.55; 1.67	<0.001
ANT Lee vs. ANT Poortmans	1.25 ± 0.10	0.93; 1.56	<0.001	0.77 ± 0.12	0.37; 1.17	<0.001	1.98 ± 0.13	1.54; 2.42	<0.001

* DXA: computed axial densitometry; BIA: bioimpedance; ANT: anthropometry; Kg: kilograms.

Table 5 shows the Bonferroni adjustment for both the general sample and the segmented sample in men and women, with respect to SMM in percentages. In the overall sample, this adjustment showed significant differences between all methods and formulas (*p* < 0.001), with the exception of DXA with anthropometry proposal of Poortmans (*p* = 1.000); BIA with anthropometry equation of Matiegka (p = 1. 000); and the anthropometry proposals of Kerr (opt. 1) with Matiegka and Heymsfield (*p* = 0.397–1.000), Kerr (opt. 2) with Lee (*p* = 1.000), Matiegka with Heymsfield (*p* = 0.351), and Drinkwater with Heymsfield (0.318). In the male group, all methods and formulas presented significant differences (*p* < 0.001–0.035), with the exception of BIA with the anthropometry equations proposed by Kerr (opt. 1) and Heymsfield (*p* = 0.312–0.393); and the anthropometry formulas of Kerr (opt. 2) with Matiegka, Drinkwater and Lee (0.167–0.257), and Matiegka with Lee and Poortmans (*p* = 0.754–1.000). In the female group, all methods and equations showed significant differences (*p* < 0.001-0.033), with the exception of DXA with Heymsfield and Poortmans anthropometry formulas (*p* = 0.341-0.482); and Kerr (opt. 1) and Lee (*p* = 1.000) anthropometry equations.

**Table 5:** Differences between methods of estimating skeletal muscle mass in percentages.

Comparison	General sample (*n* = 262)			Males (*n* = 159)			Females (*n* = 103)		
	Mean differences ± SD	95%CI (min; max)	*p-*value	Mean differences ± SD	95%CI (min; max)	*p*-value	Mean differences ± SD	95%CI (min; max)	*p-*value
DXA vs. BIA	–7.84 ± 0.21	–8.54; –7.13	<0.001	–6.14 ± 0.22	–6.88; –5.40	<0.001	–10.45 ± 0.26	–11.32; –9.59	<0.001
DXA vs. ANT Kerr (opt. 1)	–5.65 ± 0.23	–6.42; –4.88	<0.001	–5.41 ± 0.32	–6.48; –4.35	<0.001	–6.02 ± 0.32	–7.10; –4.93	<0.001
DXA vs. ANT Kerr (opt. 2)	–2.45 ± 0.24	–3.23; –1.66	<0.001	–2.73 ± 0.32	–3.79; –1.67	<0.001	–2.01 ± 0.35	–3.19; –0.38	<0.001
DXA vs. ANT Matiegka	–7.12 ± 0.53	–8.87; –5.36	<0.001	–1.39 ± 0.39	–2.68; –0.09	0.022	–15.96 ± 0.48	–17.55; –14.36	<0.001
DXA vs. ANT Martin	–13.44 ± 0.24	–14.22; –12.66	<0.001	–14.14 ± 0.32	–15.19; –13.10	<0.001	–12.36 ± 0.33	–13.473; –11.25	<0.001
DXA vs. ANT Drinkwater	–3.94 ± 0.17	–4.50; –3.37	<0.001	–3.23 ± 0.22	–3.97; –2.48	<0.001	–5.03 ± 0.23	–5.80; –4.26	<0.001
DXA vs. ANT Heymsfield	–4.98 ± 0.40	–6.29; –3.66	<0.001	–7.54 ± 0.51	–9.24; –5.83	<0.001	–1.02 ± 0.39	–2.34; 0.30	0.482
DXA vs. ANT Lee	–2.35 ± 0.18	–2.94; –1.75	<0.001	–2.16 ± 0.25	–3.00; –1.32	<0.001	–2.63 ± 0.24	–3.42; –1.85	<0.001
DXA vs. ANT Poortmans	–0.30 ± 0.22	–1.01; 0.42	1.000	–1.00 ± 0.29	–1.97; –0.03	0.035	0.80 ± 0.29	–0.19; 1.78	0.341
BIA vs. ANT Kerr (opt. 1)	2.19 ± 0.24	1.40; 2.98	<0.001	0.73 ± 0.27	–0.16; 1.61	0.312	4.44 ± 0.35	3.27; 5.61	<0.001
BIA vs. ANT Kerr (opt. 2)	5.39 ± 0.27	4.49; 6.29	<0.001	3.41 ± 0.28	2.48; 4.35	<0.001	8.44 ± 0.38	7.16; 9.72	<0.001
BIA vs. ANT Matiegka	0.72 ± 0.39	–0.58; 2.02	1.000	4.75 ± 0.27	3.86; 5.65	<0.001	–5.50 ± 0.46	–7.04; –3.97	<0.001
BIA vs. ANT Martin	–5.61 ± 0.31	–6.62; –4.59	<0.001	–8.00 ± 0.32	–9.08; –6.93	<0.001	–1.91 ± 0.38	–3.20; –0.62	<0.001
BIA vs. ANT Drinkwater	3.90 ± 0.16	3.39; 4.41	<0.001	2.91 ± 0.16	2.39; 3.44	<0.001	5.43 ± 0.24	4.61; 6.24	<0.001
BIA vs. ANT Heymsfield	2.86 ± 0.48	1.27; 4.45	<0.001	–1.40 ± 0.53	–3.14; 0.35	0.393	9.43 ± 0.39	8.12; 10.74	<0.001
BIA vs. ANT Lee	5.49 ± 0.19	4.86; 6.12	<0.001	3.98 ± 0.18	3.37; 4.59	<0.001	7.82 ± 0.26	6.94; 8.70	<0.001
BIA vs. ANT Poortmans	7.54 ± 0.27	6.64; 8.44	<0.001	5.14 ± 0.26	4.29; 5.99	<0.001	11.25 ± 0.32	10.17; 12.33	<0.001
ANT Kerr (opt. 1) vs. ANT Kerr (opt. 2)	3.20 ± 0.07	2.96; 3.45	<0.001	2.68 ± 0.08	2.43; 2.93	<0.001	4.01 ± 0.11	3.64; 4.37	<0.001
ANT Kerr (opt. 1) vs. ANT Matiegka	–1.47 ± 0.56	–3.30; 0.37	0.397	4.03 ± 0.45	2.54; 5.51	<0.001	–9.94 ± 0.61	–11.98; –7.91	<0.001
ANT Kerr (opt. 1) vs. ANT Martin	–7.79 ± 0.19	–8.41; –7.17	<0.001	–8.73 ± 0.24	–9.52; –7.74	<0.001	–6.35 ± 0.25	–7.19; –5.51	<0.001
ANT Kerr (opt. 1) vs. ANT Drinkwater	1.71 ± 0.13	1.29; 2.13	<0.001	2.18 ± 0.16	1.65; 2.72	<0.001	0.99 ± 0.18	0.37; 1.60	<0.001
ANT Kerr (opt. 1) vs. ANT Heymsfield	0.67 ± 0.34	–0.46; 1.81	1.000	–2.12 ± 0.39	–3.43; –0.81	<0.001	4.99 ± 0.31	3.96; 6.03	<0.001
ANT Kerr (opt. 1) vs. ANT Lee	3.30 ± 0.16	2.79; 3.82	<0.001	3.25 ± 0.20	2.59; 3.92	<0.001	3.38 ± 0.26	2.52; 4.24	<0.001
ANT Kerr (opt. 1) vs. ANT Poortmans	5.35 ± 0.15	4.86; 5.85	<0.001	4.41 ± 0.15	3.91; 4.91	<0.001	6.81 ± 0.24	6.02; 7.60	<0.001
ANT Kerr (opt. 2) vs. ANT Matiegka	–4.67 ± 0.60	–6.65; –2.69	<0.001	1.34 ± 0.47	–0.21; 2.90	0.211	–13.95 ± 0.65	–16.14; –11.76	<0.001
ANT Kerr (opt. 2) vs. ANT Martin	–11.00 ± 0.16	–11.52; –10.47	<0.001	–11.41 ± 0.21	–12.10; –10.73	<0.001	–10.35 ± 0.23	–11.14; –9.57	<0.001
ANT Kerr (opt. 2) vs. ANT Drinkwater	–1.49 ± 0.16	–2.00; –0.98	<0.001	–0.50 ± 0.17	–1.06; 0.06	0.167	–3.02 ± 0.22	–3.77; –2.27	<0.001
ANT Kerr (opt. 2) vs. ANT Heymsfield	–2.53 ± 0.31	–3.56; –1.50	<0.001	–4.81 ± 0.39	–6.09; –3.52	<0.001	0.99 ± 0.28	0.04; 1.94	0.033
ANT Kerr (opt. 2) vs. ANT Lee	0.10 ± 0.17	–0.46; 0.66	1.000	0.57 ± 0.20	–0.11; 1.25	0.257	–0.62 ± 0.28	–1.58; 0.33	1.000
ANT Kerr (opt. 2) vs. ANT Poortmans	2.15 ± 0.12	1.74; 2.56	<0.001	1.73 ± 0.14	1.27; 2.18	<0.001	2.81 ± 0.22	2.07; 3.54	<0.001
ANT Matiegka vs. ANT Martin	–6.33 ± 0.64	–8.43; –4.23	<0.001	–12.75 ± 0.51	–14.43; –11.08	<0.001	3.59 ± 0.66	1.37; 5.82	<0.001
ANT Matiegka vs. ANT Drinkwater	3.18 ± 0.48	1.61; 4.75	<0.001	–1.84 ± 0.35	–2.99; –0.70	<0.001	10.93 ± 0.48	9.33; 12.53	<0.001
ANT Matiegka vs. ANT Heymsfield	2.14 ± 0.80	–0.49; 4.77	0.351	–6.15 ± 0.68	–8.41; –3.89	<0.001	14.93 ± 0.63	12.83; 17.04	<0.001
ANT Matiegka vs. ANT Lee	4.77 ± 0.50	3.14; 6.40	<0.001	–0.77 ± 0.32	–1.83; 0.29	0.754	13.32 ± 0.41	11.94; 14.71	<0.001
ANT Matiegka vs. ANT Poortmans	6.82 ± 0.60	4.84; 8.80	<0.001	0.38 ± 0.43	–1.03; 1.80	1.000	16.75 ± 0.56	14.87; 18.63	<0.001
ANT Martin vs. ANT Drinkwater	9.51 ± 0.20	8.83; 10.18	<0.001	10.91 ± 0.23	10.13; 11.69	<0.001	7.33 ± 0.25	6.50; 8.17	<0.001
ANT Martin vs. ANT Heymsfield	8.47 ± 0.35	7.30; 9.64	<0.001	6.61 ± 0.47	5.03; 8.18	<0.001	11.34 ± 0.38	10.05; 12.63	<0.001
ANT Martin vs. ANT Lee	11.10 ± 0.21	10.40; 11.80	<0.001	11.98 ± 0.26	11.11; 12.85	<0.001	9.73 ± 0.31	8.68; 10.78	<0.001
ANT Martin vs. ANT Poortmans	13.15 ± 0.14	12.68; 13.62	<0.001	13.14 ± 0.18	12.53; 13.74	<0.001	13.16 ± 0.23	12.39; 13.93	<0.001
ANT Drinkwater vs. ANT Heymsfield	–1.04 ± 0.38	–2.30; 0.22	0.318	–4.31 ± 0.43	–5.74; –2.88	<0.001	4.01 ± 0.31	2.96; 5.06	<0.001
ANT Drinkwater vs. ANT Lee	1.59 ± 0.11	1.24; 1.94	<0.001	1.07 ± 0.12	0.66; 1.48	<0.001	2.39 ± 0.16	1.85; 2.94	<0.001
ANT Drinkwater vs. ANT Poortmans	3.64 ± 0.17	3.09; 4.19	<0.001	2.23 ± 0.16	1.70; 2.76	<0.001	5.83 ± 0.21	5.14; 6.51	<0.001
ANT Heymsfield vs. ANT Lee	2.63 ± 0.35	1.48; 3.78	<0.001	5.38 ± 0.41	4.00; 6.75	<0.001	–1.61 ± 0.30	–2.61; –0.61	<0.001
ANT Heymsfield vs. ANT Poortmans	4.68 ± 0.29	3.72; 5.64	<0.001	6.53 ± 0.38	5.26; 7.81	<0.001	1.82 ± 0.27	0.91; 2.73	<0.001
ANT Lee vs. ANT Poortmans	20.05 ± 0.15	1.54; 2.56	<0.001	1.16 ± 0.17	0.60; 1.71	<0.001	3.43 ± 0.24	2.64; 4.23	<0.001

* DXA, computed axial densitometry; BIA, bioimpedance; ANT, anthropometry; %: percentages.

Table 6 shows the Bonferroni adjustment for the total group, as well as for the men and women groups, with respect to LBM in kilograms and percentages. All methods and equations presented significant differences (p < 0.001-0.001), except for the LBM in kilograms of DXA with anthropometry according to Lee (*p* = 0.177) in the men’s group; and the LBM expressed in percentages of DXA and BIA (*p* = 0.394) also in the men’s group.

**Table 6:** Differences between the methods of estimating lean body mass in kg and percentages.

Comparison		General sample (*n* = 262)			Males (*n* = 159)			Females (*n* = 103)		
		Mean differences ± SD	95%CI (min; max)	*p*-value	Mean differences ± SD	95%CI (min; max)	*p-*value	Mean differences ± SD	95%CI (min; max)	*p*-value
Lean body mass (kg)	DXA vs. BIA	–1.96 ± 0.16	–2.37; –1.54	<0.001	–1.34 ± 0.21	–1.91; –0.77	<0.001	–2.91 ± 0.19	–3.42; –2.39	<0.001
	DXA vs. ANT Lee	0.72 ± 0.18	0.23; 1.21	0.001	0.57 ± 0.26	–0.12; 1.27	0.177	0.95 ± 0.24	0.31; 1.59	0.001
	DXA vs. ANT Kulkarni	–8.01 ± 0.25	–8.66; –7.36	<0.001	–9.59 ± 0.31	–10.42; –8.77	<0.001	–5.55 ± 0.26	–6.24; –4.87	<0.001
	BIA vs. ANT Lee	2.68 ± 0.14	2.29; 3.06	<0.001	1.91 ± 0.16	1.50; 2.33	<0.001	3.85 ± 0.23	3.23; 4.48	<0.001
	BIA vs. ANT Kulkarni	–6.05 ± 0.23	–6.66; –5.44	<0.001	–8.26 ± 0.20	–8.79; –7.72	<0.001	–2.65 ± 0.24	–3.29; –2.00	<0.001
	ANT Lee vs. ANT Kulkarni	8.73 ± 0.15	8.34; 9.11	<0.001	–10.17 ± 0.13	–10.50; –9.83	<0.001	–6.50 ± 0.15	–6.89; –6.11	<0.001
Lean body mass (%)	DXA vs. BIA	–1.85 ± 0.23	–2.45; –1.24	<0.001	–0.50 ± 0.27	–1.22; 0.22	0.394	–3.93 ± 0.31	–4.75; –3.10	<0.001
	DXA vs. ANT Lee	2.14 ± 0.25	1.48; 2.80	<0.001	1.98 ± 0.32	1.12; 2.84	<0.001	2.39 ± 0.40	1.32; 3.46	<0.001
	DXA vs. ANT Kulkarni	–9.96 ± 0.28	–10.72; –9.21	<0.001	–11.09 ± 0.37	–12.09; –10.09	<0.001	–8.22 ± 0.38	–9.25; –7.20	<0.001
	BIA vs. ANT Lee	3.99 ± 0.22	3.39; 4.58	<0.001	2.48 ± 0.20	1.95; 3.01	<0.001	6.31 ± 0.38	5.30; 7.33	<0.001
	BIA vs. ANT Kulkarni	–8.12 ± 0.27	–8.84; –7.39	<0.001	–10.59 ± 0.22	–11.17; –10.1	<0.001	–4.30 ± 0.37	–5.29; –3.31	<0.001
	ANT Lee vs. ANT Kulkarni	–12.10 ± 0.12	–12.42; –11.79	<0.001	–13.07 ± 0.14	–13.43; –12.71	<0.001	–10.61 ± 0.11	–10.91; –10.31	<0.001

* DXA, computed axial densitometry; BIA, bioimpedance; ANT, anthropometry; %: percentages.

### 3.2 Agreement and concordance with reference method (DXA)

Lin’s Concordance Correlation Coefficient (CCC) was used to evaluate the concordance between each estimation method and the reference method, DXA. The findings are presented in Table 7 and Supplementary Figures S1–S9. Supplementary Figures S1–S3 show SMM in kilograms for the general sample, males and females, respectively. Supplementary Figures S4–S6 display SMM in percentages for the same groups. Supplementary Figures S7–S9 present LBM in both kilograms and percentages for the general sample, males, and females, respectively.

**Table 7:** Lin’s Concordance Correlation Coefficient for the agreement between methods of estimating skeletal muscle mass and lean body mass for the overall sample and divided by sex.

Variable	Methods	General sample (*n* = 262)			Male sample (*n* = 159)			Female sample (*n* = 103)		
		CCC	ρ	Cb	CCC	ρ	Cb	CCC	ρ	Cb
SMM (kg)	DXA vs. BIA	0.690	0.957	0.721	0.539	0.883	0.610	0.244	0.833	0.293
	DXA vs. ANT Kerr (opt. 1)	0.813	0.930	0.874	0.592	0.792	0.747	0.455	0.784	0.581
	DXA vs. ANT Kerr (opt. 2)	0.910	0.935	0.973	0.735	0.804	0.915	0.696	0.779	0.895
	DXA vs. ANT Lee et al.	0.930	0.961	0.968	0.775	0.853	0.908	0.675	0.838	0.806
	DXA vs. ANT Poortmans	0.941	0.946	0.995	0.812	0.824	0.985	0.777	0.793	0.980
	DXA vs. ANT Matiegka	0.334	0.918	0.363	0.418	0.791	0.529	0.0450	0.684	0.066
	DXA vs. ANT Martin et al.	0.587	0.945	0.622	0.302	0.838	0.361	0.254	0.843	0.302
	DXA vs. ANT Drinkwater y Ross	0.880	0.961	0.915	0.757	0.877	0.862	0.519	0.844	0.615
	DXA vs. ANT Heymsfield et al.	0.848	0.927	0.915	0.514	0.754	0.682	0.682	0.710	0.961
SMM (%)	DXA vs. BIA	0.341	0.863	0.395	0.317	0.820	0.387	0.166	0.792	0.209
	DXA vs. ANT Kerr (opt. 1)	0.498	0.794	0.627	0.248	0.522	0.476	0.209	0.581	0.359
	DXA vs. ANT Kerr (opt. 2)	0.721	0.789	0.913	0.420	0.541	0.777	0.420	0.513	0.820
	DXA vs. ANT Lee et al.	0.804	0.884	0.910	0.618	0.731	0.844	0.643	0.802	0.801
	DXA vs. ANT Poortmans	0.845	0.846	0.998	0.609	0.645	0.945	0.675	0.690	0.978
	DXA vs. ANT Matiegka	0.007	0.011	0.578	0.433	0.453	0.957	0.103	0.620	0.166
	DXA vs. ANT Martin et al.	0.2691	0.833	0.323	0.118	0.639	0.185	0.118	0.661	0.178
	DXA vs. ANT Drinkwater y Ross	0.654	0.907	0.721	0.538	0.802	0.672	0.358	0.816	0.439
	DXA vs. ANT Heymsfield et al.	0.648	0.785	0.826	0.287	0.510	0.564	0.557	0.574	0.971
LBM (kg)	DXA vs. BIA	0.957	0.981	0.976	0.913	0.943	0.969	0.797	0.934	0.854
	DXA vs. ANT Kulkarni	0.813	0.964	0.843	0.489	0.882	0.554	0.540	0.881	0.613
	DXA vs. ANT Lee	0.969	0.971	0.998	0.905	0.916	0.988	0.850	0.877	0.96
LBM (%)	DXA vs. BIA	0.847	0.907	0.935	0.815	0.855	0.952	0.710	0.869	0.817
	DXA vs. ANT Kulkarni	0.474	0.841	0.564	0.173	0.706	0.245	0.289	0.767	0.377
	DXA vs. ANT Lee	0.838	0.874	0.958	0.669	0.815	0.821	0.656	0.742	0.885

*CCC, Concordance Correlation Coefficient; Cb, Bias correlation factor; SMM, Skeletal muscle mass; LBM, Lean body mass; DXA, computed axial densitometry; BIA, bioimpedance; ANT, anthropometry; Kg, kilograms, %, percentages.

Overall, the analysis reveals poor concordance among the estimation methods and DXA (CCC = 0.007–0.880), as shown in Supplementary Figure S1–S9. Notable exceptions include moderate concordance between DXA and the anthropometry formulas of Kerr opt 2, Lee, and Poortmans for SMM in kilograms in the general sample (CCC = 0.910; 0.941) (Supplementary Figure S1). There is also a substantial concordance between DXA and BIA and DXA and Lee’s anthropometry formula for LBM in kilograms in the general sample (CCC = 0.957; 0.969) (Supplementary Figure S7). Additionally, moderate to substantial concordance is noted for the general sample and the male subgroup between DXA and BIA, and DXA and Lee’s anthropometry formula for LBM in kilograms (CCC = 0.913; 0.905-0.969), as shown in Supplementary Figure S8.

The Bland–Altman analysis was employed to evaluate the agreement between each estimation method and the reference method, DXA. Table 8 illustrates the results of this analysis for SMM and LBM in both kilograms and percentages for the overall sample and separated by sex. The findings are presented in Supplementary Figures S10–S18. Specifically, Supplementary Figure S10–S12 show SMM in kilograms for the general sample, males and females, respectively. Supplementary Figures S13–S15 display SMM in percentages for the general sample, males and females, respectively Supplementary Figures S16–S18 show LBM for the general sample, males and females, respectively. The Bland–Altman analysis revealed that most methods and equations did not show agreement with DXA for SMM (*p* < 0.001-0.023). Similarly, for LBM, most methods and equations did not show agreement with DXA (p < 0.001-0.029). However, the Poortmans anthropometric equation for SMM in percentages in the general sample showed agreement with DXA (*p* = 0.177) (Supplementary Figure S13). For LBM, BIA in percentages in the male group also showed agreement with DXA (*p* = 0.066) (Supplementary Figure S17).

**Table 8:** Bland–Altman Plot for the agreement between methods of estimating skeletal muscle mass and lean body mass for the overall sample and divided by sex.

	Method	Pearson’s *r* (p)							
Variable			Mean diff	95% CI	95% Limits of agreement		p		
					Lower limit	Upper limit		Regression equation	p
SMM (kg)	GENERAL SAMPLE (n = 262)								
	DXA vs. BIA	0.954 (*p* < 0.001)	–5.404	–5.677 to–5.131	–9.804	–1.004	<0.001	*y* = –8.837 + 0.117 x	<0.001
	DXA vs. ANT Kerr (opt. 1)	0.926 (*p* < 0.001)	–4.186	–4.561 to–3.820	–10.238	1.867	<0.001	*y* = –1.054 + –0.109 x	<0.001
	DXA vs. ANT Kerr (opt. 2)	0.932 (*p* < 0.001)	–1.961	–2.323 to–1.599	–7.796	3.874	<0.001	*y* = 1.224 + –0.115 x	<0.001
	DXA vs. ANT Matiegka	0.913 (*p* < 0.001)	–4.146	–4.789 to–3.504	–14.498	6.206	<0.001	*y* = –34.882 + 1.066 x	<0.001
	DXA vs. ANT Martin	0.940 (*p* < 0.001)	–9.853	–10.336 to–9.370	–17.639	–2.067	<0.001	*y* = 0.157 + –0.316 x	<0.001
	DXA vs. ANT Drinkwater	0.959 (*p* < 0.001)	–2.798	–3.050 to–2.546	–6.857	1.261	<0.001	*y* = –3.650 + 0.030 x	0.092
	DXA vs. ANT Heymsfield	0.912 (*p* < 0.001)	–3.963	–4.593 to–3.332	–14.121	6.196	<0.001	*y* = 7.802 + –0.409 x	<0.001
	DXA vs. ANT Lee	0.956 (*p* < 0.001)	–1.615	–1.877 to–1.353	–5.832	2.602	<0.001	*y* = –2.625 + 0.037 x	0.050
	DXA vs. ANT Poortmans	0.946 (*p* < 0.001)	–0.369	–0.686 to–0.0512	–5.4817	4.745	0.023	*y* = 1.995 + –0.088 x	<0.001
	MALES (n = 159)								
	DXA vs. BIA	0.885 (*p* < 0.001)	–4.775	–5.126 to–4.423	–9.174	–0.376	<0.001	*y* = –9.908 + 0.151 x	<0.001
	DXA vs. ANT Kerr (opt. 1)	0.800 (*p* < 0.001)	–4.408	–4.947 to–3.868	–11.156	2.341	<0.001	*y* = 1.980 + –0.189 x	0.001
	DXA vs. ANT Kerr (opt. 2)	0.811 (*p* < 0.001)	–2.312	–2.826 to–1.797	–8.749	4.125	<0.001	*y* = 3.050 + –0.163 x	0.002
	DXA vs. ANT Matiegka	0.790 (*p* < 0.001)	–0.754	–1.340 to–0.167	–8.093	6.586	0.0121	*y* = –36.953 + 1.130 x	<0.001
	DXA vs. ANT Martin	0.838 (*p* < 0.001)	–11.238	–11.862 to–10.615	–19.040	–3.436	<0.001	*y* = 3.824 + –0.404 x	<0.001
	DXA vs. ANT Drinkwater	0.883 (*p* < 0.001)	–2.580	–2.941 to–2.219	–7.098	1.938	<0.001	*y* = –3.568 + 0.030 x	0.452
	DXA vs. ANT Heymsfield	0.749 (*p* < 0.001)	–6.081	–6.925 to–5.236	–16.647	4.486	<0.001	*y* = 13.117 + –0.553 x	<0.001
	DXA vs. ANT Lee	0.851 (*p* < 0.001)	–1.648	–2.045 to–1.252	–6.610	3.314	<0.001	*y* = –7.042 + 0.166 x	<0.001
	DXA vs. ANT Poortmans	0.824 (*p* < 0.001)	–0.876	–1.336 to–0.417	–6.627	4.875	<0.001	*y* = 0.642 + –0.047 x	0.342
	FEMALES (n = 103)								
	DXA vs. BIA	0.817 (*p* < 0.001)	–6.375	–6.741 to–6.008	–10.054	–2.695	<0.001	*y* = –1.102 + –0.235 x	<0.001
	DXA vs. ANT Kerr (opt. 1)	0.800 (*p* < 0.001)	–3.843	–4.315 to–3.370	–8.580	0.895	<0.001	*y* = 5.494 + –0.442 x	<0.001
	DXA vs. ANT Kerr (opt. 2)	0.797 (*p* < 0.001)	–1.419	–1.877 to–0.962	–6.009	3.170	<0.001	*y* = 6.766 + –0.411 x	<0.001
	DXA vs. ANT Matiegka	0.671 (*p* < 0.001)	–9.383	–9.775 to–8.992	–13.313	–5.454	<0.001	*y* = –28.684 + 0.807 x	<0.001
	DXA vs. ANT Martin	0.856 (*p* < 0.001)	–7.715	–8.276 to–7.155	–13.338	–2.093	<0.001	*y* = 6.641 + –0.622 x	<0.001
	DXA vs. ANT Drinkwater	0.850 (*p* < 0.001)	–3.134	–3.447 to–2.822	–6.266	–0.002	<0.001	*y* = 0.113 + –0.156 x	0.007
	DXA vs. ANT Heymsfield	0.723 (*p* < 0.001)	–0.693	–1.169 to–0.217	–5.465	4.080	0.0047	*y* = 5.921 + –0.338 x	<0.001
	DXA vs. ANT Lee	0.852 (*p* < 0.001)	–1.564	–1.834 to–1.294	–4.275	1.147	<0.001	*y* = –3.709 + 0.107 x	<0.001
	DXA vs. ANT Poortmans	0.807 (*p* < 0.001)	0.416	0.0740 to 0.757	–3.010	3.841	0.018	*y* = 2.692 + –0.120 x	0.068
SMM (%)	GENERAL SAMPLE (n = 262)								
	DXA vs. BIA	0.853 (*p* < 0.001)	–7.836	–8.256 to–7.416	–14.600	–1.071	<0.001	*y* = –26.785 + 0.462 x	<0.001
	DXA vs. ANT Kerr (opt. 1)	0.792 (*p* < 0.001)	–5.649	–6.107 to–5.191	–13.025	1.727	<0.001	*y* = –14.130 + 0.212 x	<0.001
	DXA vs. ANT Kerr (opt. 2)	0.792 (*p* < 0.001)	–2.446	–2.917 to–1.976	–10.020	5.127	<0.001	*y* = –4.950 + 0.065 x	0.123
	DXA vs. ANT Matiegka	–0.014 (*p* = 0.817)	–7.115	–8.166 to–6.064	–24.043	9.813	<0.001	*y* = –9.496 + 0.059 x	0.642
	DXA vs. ANT Martin	0.831 (*p* < 0.001)	–13.442	–13.908 to–12.975	–20.954	–5.929	<0.001	*y* = –8.293 + –0.117 x	0.002
	DXA vs. ANT Drinkwater	0.907 (*p* < 0.001)	–3.936	–4.275 to–3.598	–9.3894	1.517	<0.001	*y* = –15.978 + 0.308 x	<0.001
	DXA vs. ANT Heymsfield	0.767 (*p* < 0.001)	–4.975	–5.762 to–4.188	–17.652	7.702	<0.001	*y* = 15.577 + –0.519 x	<0.001
	DXA vs. ANT Lee	0.881 (*p* < 0.001)	–2.345	–2.698 to–1.992	–8.039	3.349	<0.001	*y* = –7.010 + 0.122 x	<0.001
	DXA vs. ANT Poortmans	0.842 (*p* < 0.001)	–0.295	–0.725 to 0.134	–7.213	6.623	0.177	*y* = 1.214 + –0.0405 x	0.266
	MALES (n = 159)								
	DXA vs. BIA	0.821 (*p* < 0.001)	–6.140	–6.580 to–5.701	–11.640	–0.641	<0.001	*y* = –27.381 + 0.486 x	<0.001
	DXA vs. ANT Kerr (opt. 1)	0.529 (*p* < 0.001)	–5.412	–6.046 to–4.779	–13.340	2.516	<0.001	*y* = –25.129 + 0.455 x	<0.001
	DXA vs. ANT Kerr (opt. 2)	0.551 (*p* < 0.001)	–2.729	–3.362 to–2.097	–10.644	5.186	<0.001	*y* = –16.310 + 0.324 x	0.0002
	DXA vs. ANT Matiegka	0.443 (*p* < 0.001)	–1.387	–2.158 to–0.616	–11.029	8.255	0.001	*y* = –1.673 + 0.007 x	0.944
	DXA vs. ANT Martin	0.641 (*p* < 0.001)	–14.141	–14.762 to–13.519	–21.917	–6.365	<0.001	*y* = –13.973 + –0.004 x	<0.001
	DXA vs. ANT Drinkwater	0.806 (*p* < 0.001)	–3.229	–3.672 to–2.786	–8.774	2.317	<0.001	*y* = –21.112 + 0.424 x	<0.001
	DXA vs. ANT Heymsfield	0.492 (*p* < 0.001)	–7.535	–8.551 to–6.520	–20.243	5.172	<0.001	*y* = 18.498 + –0.587 x	<0.001
	DXA vs. ANT Lee	0.732 (*p* < 0.001)	–2.158	–2.658 to–1.658	–8.415	4.099	<0.001	*y* = –13.655 + 0.276 x	<0.001
	DXA vs. ANT Poortmans	0.639 (*p* < 0.001)	–1.003	–1.580 to–0.425	–8.233	6.228	0.001	*y* = –11.015 + 0.244 x	0.001
	FEMALES (n = 103)								
	DXA vs. BIA	0.772 (*p* < 0.001)	–10.453	–10.966 to–9.940	–15.597	–5.309	<0.001	*y* = –11.998 + 0.042 x	0.560
	DXA vs. ANT Kerr (opt. 1)	0.583 (*p* < 0.001)	–6.015	–6.653 to–5.376	–12.419	0.389	<0.001	*y* = –19.515 + 0.388 x	<0.001
	DXA vs. ANT Kerr (opt. 2)	0.525 (*p* < 0.001)	–2.010	–2.704 to–1.315	–8.975	4.955	<0.001	*y* = –10.862 + 0.270 x	0.016
	DXA vs. ANT Matiegka	0.615 (*p* < 0.001)	–15.957	–16.900 to–15.014	–25.417	–6.497	<0.001	*y* = 4.977 + –0.526 x	<0.001
	DXA vs. ANT Martin	0.662 (*p* < 0.001)	–12.363	–13.019 to–11.706	–18.943	–5.782	<0.001	*y* = –9.669 + –0.071 x	0.431
	DXA vs. ANT Drinkwater	0.814 (*p* < 0.001)	–5.028	–5.484 to–4.573	–9.593	–0.464	<0.001	*y* = –17.369 + 0.360 x	<0.001
	DXA vs. ANT Heymsfield	0.564 (*p* < 0.001)	–1.023	–1.803 to–0.242	–8.847	6.802	0.011	*y* = 4.569 + –0.173 x	0.101
	DXA vs. ANT Lee	0.808 (*p* < 0.001)	–2.634	–3.099 to–2.169	–7.301	2.033	<0.001	*y* = –5.045 + 0.073 x	0.263
	DXA vs. ANT Poortmans	0.695 (*p* < 0.001)	0.797	0.217 to 1.377	–5.023	6.617	0.008	*y* = –2.705 + 0.112 x	0.189
LBM (kg)	GENERAL SAMPLE (n = 262)								
	DXA vs. BIA	0.979 (*p* < 0.001)	–1.956	–2.265 to–1.646	–6.943	3.032	<0.001	*y* = –6.600 + 0.088 x	<0.001
	DXA vs. ANT Kulkarni	0.959 (*p* < 0.001)	–8.006	–8.488 to–7.524	–15.770	–0.241	<0.001	*y* = –1.065 + –0.124 x	<0.001
	DXA vs. ANT Lee	0.969 (*p* < 0.001)	0.720	0.358 to 1.081	–5.108	6.547	<0.001	*y* = –1.078 + 0.035 x	0.026
	MALES (n = 159)								
	DXA vs. BIA	0.945 (*p* < 0.001)	–1.339	–1.762 to–0.916	–6.636	3.958	<0.001	*y* = –9.883 + 0.141 x	<0.001
	DXA vs. ANT Kulkarni	0.879 (*p* < 0.001)	–9.594	–10.203 to–8.985	–17.215	–1.972	<0.001	*y* = –13.617 + 0.062 x	0.126
	DXA vs. ANT Lee	0.916 (*p* < 0.001)	0.572	0.0578 to 1.087	–5.867	7.012	0.030	*y* = –8.918 + 0.159 x	<0.001
	FEMALES (n = 103)								
	DXA vs. BIA	0.927 (*p* < 0.001)	–2.907	–3.286 to–2.529	–6.703	0.889	<0.001	*y* = –0.960 + –0.047 x	0.226
	DXA vs. ANT Kulkarni	0.875 (*p* < 0.001)	–5.554	–6.061 to–5.047	–10.641	–0.467	<0.001	*y* = –1.946 + –0.085 x	0.102
	DXA vs. ANT Lee	0.873 (*p* < 0.001)	0.947	0.476 to 1.418	–3.779	5.673	<0.001	*y* = –6.193 + 0.181 x	0.001
LBM (%)	GENERAL SAMPLE (n = 262)								
	DXA vs. BIA	0.903 (*p* < 0.001)	–1.845	–2.293 to–1.397	–9.064	5.3730	<0.001	*y* = –20.118 + 0.246 x	<0.001
	DXA vs. ANT Kulkarni	0.839 (*p* < 0.001)	–9.962	–10.521 to–9.403	–18.970	–0.953	<0.001	*y* = –15.018 + 0.064 x	<0.001
	DXA vs. ANT Lee	0.874 (*p* < 0.001)	2.140	1.648 to 2.632	–5.785	10.065	<0.001	*y* = –11.441 + 0.188 x	<0.001
	MALES (n = 159)								
	DXA vs. BIA	0.855 (*p* < 0.001)	–0.498	–1.028 to 0.032	–7.136	6.140	0.066	*y* = –23.130 + 0.290 x	<0.001
	DXA vs. ANT Kulkarni	0.704 (*p* < 0.001)	–11.088	–11.824 to–10.353	–20.289	–1.888	<0.001	*y* = –68.966 + 0.693 x	<0.001
	DXA vs. ANT Lee	0.812 (*p* < 0.001)	1.981	1.346 to 2.616	–5.967	9.929	<0.001	*y* = –43.532 + 0.592 x	<0.001
	FEMALES (n = 103)								
	DXA vs. BIA	0.860 (*p* < 0.001)	–3.925	–4.535 to–3.315	–10.042	2.192	<0.001	*y* = –3.012 + –0.0133 x	0.808
	DXA vs. ANT Kulkarni	0.758 (*p* < 0.001)	–8.223	–8.977 to–7.468	–15.785	–0.660	<0.001	*y* = –38.960 + 0.434 x	<0.001
	DXA vs. ANT Lee	0.727 (*p* < 0.001)	2.386	1.598 to 3.174	–5.517	10.290	<0.001	*y* = –19.048 + 0.327 x	<0.001

SMM, Skeletal muscle mass; LBM, Lean body mass; DXA, computed axial densitometry; BIA, bioimpedance; ANT, anthropometry; Kg, kilograms, %, percentages.

Using the DXA values as a reference, all methods consistently showed a tendency to overestimate SMM in both kilograms and percentages compared to DXA in the general sample, as well as in the sex-specific groups (*r* = –0.014, *p* < 0.001-0.959, *p* < 0.001). For LBM, in both the general sample and when divided by sex, the methods that tended to overestimate compared to DXA were BIA (*r* = 0.855, *p* < 0.001 – *r* = 0.979, *p* < 0.001) and the Kulkarni anthropometric equation (*r* = 0.704, *p* < 0.001 – *r* = 0.959, *p* < 0.001). Conversely, the Lee anthropometric equation showed underestimation of the DXA results in both kilograms and percentages (*r* = 0.727, *p* < 0.001 – *r* = 0.969, *p* < 0.001). These findings are presented in Table 8.

## 4 Discussion

The main objective of this research was to evaluate the differences between DXA, BIA and anthropometry in the estimation of SMM and LBM. Firstly, significant emphasis is placed on accurately differentiating between SMM and LBM. Results obtained demonstrate notable disparities between the two metrics, with SMM yielding mean values ranging from 26.77 to 36.62 kilograms and from 37.15 to 50.59 in percentages, while LBM shows higher values between 51.27 and 59.99 kilograms and from 71.37 to 83.47 in percentages. These findings underscore the necessity of employing precise terminology in body composition assessments. Specifically, SMM yields lower values as it exclusively accounts for muscle tissue, whereas LBM includes all non-fat components, which are more abundant in the body (11). This study’s findings align with the levels of analysis according to Wang’s classification, which emphasizes the molecular and tissue-based perspectives in assessing body composition (11). These distinctions are crucial for accurate interpretations and decision-making in clinical and research settings.

The main finding of the current study was that significant differences were found between the formulas and methods used for both SMM and LBM. Previous research has revealed discrepancies when comparing different SMM formulas in anthropometry (25, 32), when comparing LBM estimation by DXA and BIA (26), and when comparing SMM results with DXA and various anthropometry equations (23, 27–29). One of the reasons for this could be the intrinsic characteristics of each method. DXA directly operates at the molecular level 2 using X-rays, estimating tissues at level 4 through regression equations based on magnetic resonance imaging as criteria (11, 17, 47). BIA is based on the differences in the conductivity properties of different tissues, estimating molecules at level 2 through estimation equations and tissues at level 4 through similar equations based on magnetic resonance imaging as reference (21, 67). Furthermore, BIA is influenced by tissue water content, leading to measurement variability (11, 12, 21). For its part, anthropometry using simple tools and indirectly estimates tissues at level 4 and molecules at level 2 through various equations that have been validated in different populations and using different methods as a reference (11, 24, 25). Discrepancies in results may arise from differences in the action principles and operational levels of DXA, BIA, and anthropometry (11, 12).

Bonferroni analysis unveiled notable discrepancies even within the same method, specifically anthropometry. This phenomenon could be elucidated by the variations encountered based on the criteria employed by each author in the development of their respective formulas (24, 25). Within this study, the discrepancies observed in the equations used for estimating SMM and LBM with anthropometry could be due, in part, to the fact that most of these formulas were validated for use in a specific population (6, 30, 31, 55–60), although the scarcity of formulas adapted to specific populations usually leads to their application in diverse contexts, including differences in sex, levels of physical activity, ethnicity, nutritional habits, and genetic factors, among others (68–70). In addition, there is a great diversity of methods used to validate the equations. While the anthropometric equations to estimate SMM by Matiegka, Kerr, and Martin used cadaver data as the reference, Poortmans validated his proposal with DXA, Heymsfield used computed axial tomography (CT), and Lee used magnetic resonance imaging (MRI) (30, 31, 56–58, 60). Not surprisingly, in light of these data, the Poortmans equation was the only anthropometric SMM approach that did not show significant differences compared to DXA.

Another objective of the present investigation was to analyze whether sex or hydration affected the comparability between methods and formulas. Sex was found to be a factor with significant influence on the comparability between methods and formulas. This could be because many equations and methods traditionally used to estimate SMM and LBM development have been used interchangeably in both sexes, irrespective of the sex for which they were validated, despite the influence of sexual dimorphism on this aspect (31, 71). Morphological divergences between males and females (72), as well as variations in bone mineralization and body water levels between sexes, exert a direct influence on estimates of body composition through methods such as DXA, BIA, and anthropometry, affecting the comparability and agreement of these instruments (34, 72–74). Therefore, sex is an essential factor to consider when using DXA, BIA, or anthropometry to assess SMM and LBM development, highlighting the imperative need to address this dimension in future research.

A relevant result of the present investigation was that hydration status did not affect the agreement between methods and formulas. This could be a consequence of the overall sample was predominantly in a well-hydrated state, although there was some variability (46).The results contrast with those found in previous studies, where it was pointed out that hydration status could have a great influence on the results found in body composition analysis, especially when assessed with BIA, as this technique is based on the evaluation of electrical conductivity, a property that is directly affected by the degree of hydration (75). It has also been suggested that hydration status should be controlled when performing DXA body composition assessments (76). Along the same lines, it has also been found that the degree of hydration could affect the results reported in the assessment of body composition with anthropometry (22). So, when assessing SMM/LBM under different hydration statuses, such as hypo-or hyper-hydration, estimates may be significantly affected (22, 76). Specifically, during hypo-hydration, where there is a deficit of body fluids, there may be a slowing down in the electrical conductivity, which may result in a BIA may overestimate body fat and underestimate lean mass or skeletal muscle mass, due to the decreased tissue water content affecting electrical conductivity (77). Conversely, in a state of hyper-hydration, where there is an excess of body fluids, BIA might overestimate lean mass and skeletal muscle mass while underestimating body fat due to increased water content which will improve electrical conductivity (77). In contrast, methods such as DXA and anthropometry are generally less sensitive to these fluctuations in hydration status and may provide more stable estimates (22, 78). This is supported by research indicating that methods such as BIA may exhibit greater variability in measurements due to their high sensitivity to changes in tissue water content (22). The fact that the present research did not show an influence of hydration status on the comparability between methods could be due to the fact that current study controlled for the main factors that could affect hydration status, such as the practice of physical exercise, the consumption of products with diuretic properties, eating heavy meals, having an injury, taking hormonal or corticosteroid treatment, or the timing of the menstrual cycle in the case of women (79–82). Therefore, it is possible that the hydration conditions of the current sample were standardized, preventing hydration status from influencing the agreement between methods and formulas. However, care should be taken when interpreting these results if standard measurement procedures are not followed to estimate SMM/LBM, especially in relation to factors that may affect hydration. It is expected that under different hydration statuses (hypo-or hyper-hydration), the estimations of SMM/LBM might be affected differently among the methods. BIA, in particular, might provide more variable data due to its high sensitivity to tissue water content (12, 22, 77, 78). Future research should test these results without controlling for factors that could affect hydration.

The second objective was to assess the agreement of BIA and anthropometry in comparison with DXA. The latter was chosen as the reference, due to its high accuracy and reliability (*r*^2^ = 0.996), and low measurement variability (coefficient of variation 0.5–4%), following the trend set by previous studies (12, 83–85). In the analysis of correlation and concordance using Lin’s concordance coefficient between various methods and the reference method, DXA, there is a general lack of concordance in most cases. This discrepancy could be due to differences in evaluation methods and the populations used for validation (6, 24, 30, 31, 55, 56, 60, 62, 63, 86). For example, some anthropometric methods measure different body parts and use various populations, which can affect the accuracy and validity of SMM and LBM estimates (6, 24, 30, 31, 55, 56, 60, 62, 63, 86).

However, there are notable exceptions. Lee, Poortmans, and Kerr (option 2, estimation with mid-thigh girth) show concordance with DXA in estimating SMM. This could be because Lee’s anthropometric SMM estimation formula was validated using nuclear magnetic resonance imaging as a reference method, and also the formula of Kim et al. that has been used in the present investigation to estimate SMM with DXA was validated using nuclear magnetic resonance imaging as a reference (17, 56). In the case of Poortmans, this formula was validated directly with DXA, which might explain the concordance (57). Respect to Kerr’s case, the concordance could be due to multiple factors, including the use of a wide range of variables in the estimation, a large sample size, and cadaver validation, which makes it an indirect method, rather than a double indirect method like the other anthropometric equations, which could minimize errors and align results with DXA (30).

For LBM, most methods also show a lack of concordance, except in the general group expressed in kilograms. Both BIA and DXA showed concordance when the sample is analyzed as a group. This pattern has also been observed in earlier studies, where BIA and DXA exhibit concordance in general samples, but not in individual cases (87). Significant concordance was also found between DXA and Lee’s formula for LBM in kg. The fact that Lee’s formula was validated using DXA as a reference may explain the concordance between the results found by both methods (6).

Another relevant finding of this research was that the Bland–Altman analysis revealed significant differences in the estimation of SMM and LBM across all methods and formulas, except for the Poortmans’s anthropometric proposal for SMM in the general sample. These differences can be partly attributed to the fact that most proposals for estimating SMM and LBM with anthropometry are indirect or doubly indirect (56, 57, 60, 88, 89), and often use validation methods other than DXA (31, 56, 58–60). However, the proposals by Poortmans for SMM, although using a doubly indirect approach, validates the equation using DXA as a reference, which could explain the similarities in the results with this reference method (6, 57).

Regarding the comparison between DXA and BIA, it was found that in most cases, BIA did not agree with the results showed by DXA. This could be due to discrepancies in the measurement principles of DXA and BIA (12, 22, 83, 90, 91). These divergences contribute to disparities in the sensitivity of the two methods to various physiological factors, which may influence the observed differences in the results obtained.

With regard to the strengths of the present research, to date, no previous study has addressed the comparison of results in the estimation of SMM and LBM using the three main methods of body composition assessment (DXA, BIA, and anthropometry), with a broad set of formulas; in such a large sample; without mixing methods that estimate SMM and LBM; and analyzing the influence of sex on the results found. Therefore, the present study has important implications for health professionals, such as nutritionists, physiotherapists and any other health professional interested in assessing SMM and/or LBM levels in their patients or athletes; as well as for sports professionals. The first is the realization that the formulas and methods are not interchangeable, and that neither BIA nor anthropometry are in agreement with DXA, except when using the Poortmans’s SMM formula with anthropometry. Therefore, given that there are differences between the results found by most methods, it is necessary, in both clinical and research body composition assessment, to consistently use the same method and formula when monitoring changes in a patient’s/user’s SMM or LBM over time, so that the measurements can be compared. Likewise, when seeking to compare an athlete to the SMM or LBM references for their discipline, or an individual to the SMM or LBM references for a chronic disease, it is imperative that the practitioner ensures that the same formula and method is used as was employed in the baseline study for the generation of the reference values. The second practical application derived from the present study is that given the identified significant influence of sex on the assessment of SMM and LBM, it is necessary to consider this factor when choosing a method and equation for assessment.

However, this study also has certain limitations that need to be addressed. Firstly, formulas were used that were not specifically validated for the population analyzed or its context, physical exercise, age and sex. However, given that this practice is common in both clinical and research settings (68–70), the decision was made to follow the same trend in the present study in order to have a record of what occurs when doing this in the researcher/clinical setting. Another limitation relates to the use of a regression formula to transform DXA-estimated LBM into SMM (17). However, this strategy was adopted, as DXA does not directly provide SMM values, in order to assess whether unifying all methods and formulas under the same level of approach to body composition (28, 29, 83), maintained or varied the observed differences. Kim’s formula 2002 (17) was chosen as it was the most popularly used in previous studies (17, 23, 27, 28, 57, 61). However, this article did not include other alternatives for estimating the SMM with DXA such as the one proposed by Kim 2004 (92) as it is less used in the literature, but future studies could also analyze the agreement with this formula.

Furthermore, regarding BIA, it was conducted in a standing position using a model that did not provide all the electrical properties in its report. Consequently, it has not been possible to calculate SMM and LBM using different bioelectrical formulas for bioimpedance in this work, as was done for anthropometry and as has been suggested in previous studies with BIA (93).

In conclusion, most of the formulas and methods used to estimate SMM and LBM with BIA and anthropometry do not show agreement nor concordance with respect to DXA, with sex having an influence on this issue. However, from the present investigation it can be extracted that for a group analysis, Poortmans, Lee and Kerr (with mid-thigh girth) anthropometry formulas for the SMM; and BIA and Lee’s anthropometry formula for the LBM showed concordance when the results were compared to DXA. For the individual analysis, only Portmans’s anthropometry formula was found to be valid with respect to DXA.

## Data Availability

The raw data supporting the conclusions of this article will be made available by the authors, without undue reservation.
