# Supplementary figures and images for: Assessing skeletal muscle mass and lean body mass: an analysis of the agreement among dual X-ray absorptiometry, anthropometry, and bioelectrical impedance
Source: Front Nutr. 2024 Aug 19;11:1445892. doi: 10.3389/fnut.2024.1445892 (PMC11366593; doi:10.3389/fnut.2024.1445892)

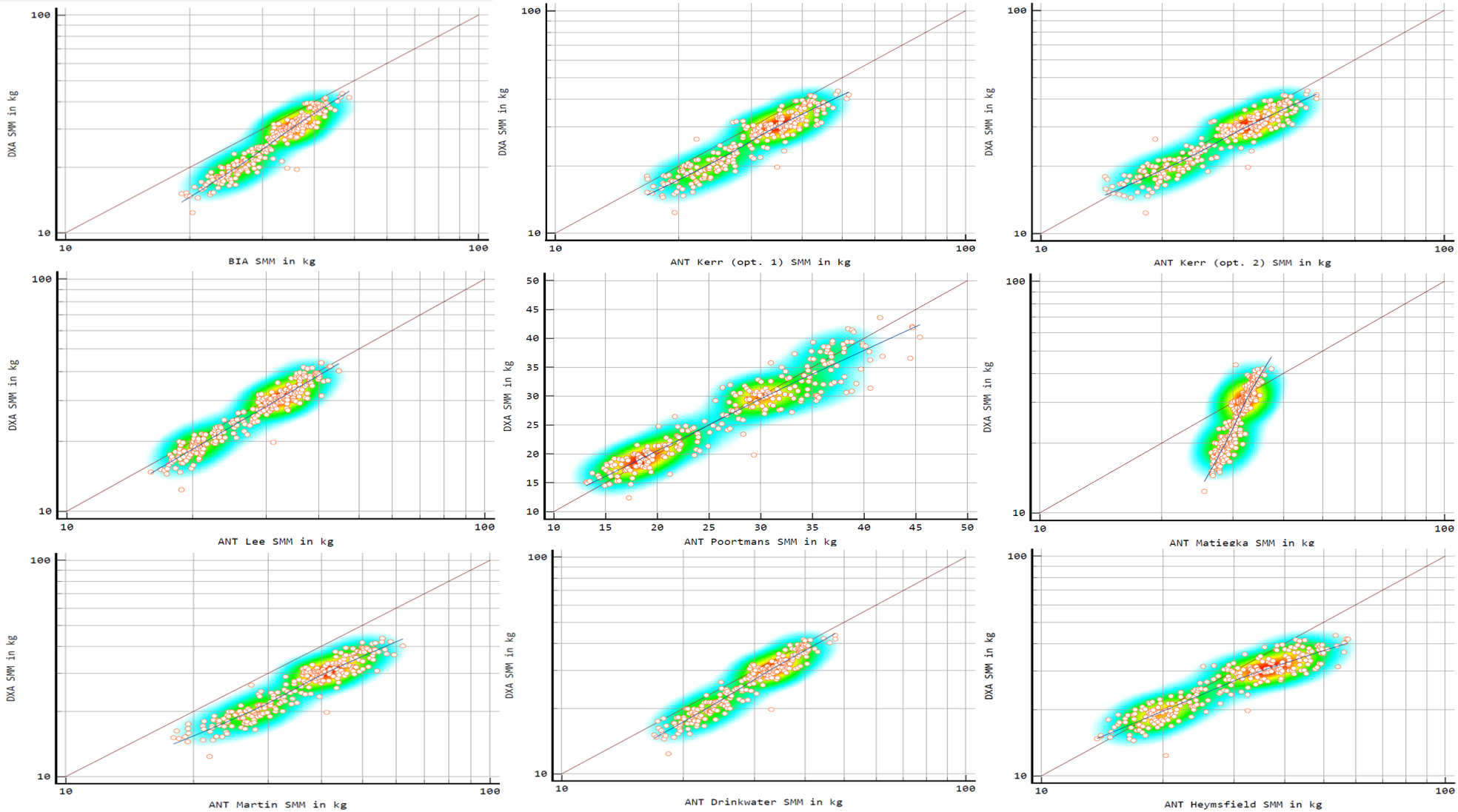

Supplement: Supplementary file 1 [file Data_Sheet_1.ZIP › Supplementary Figure 1.png]

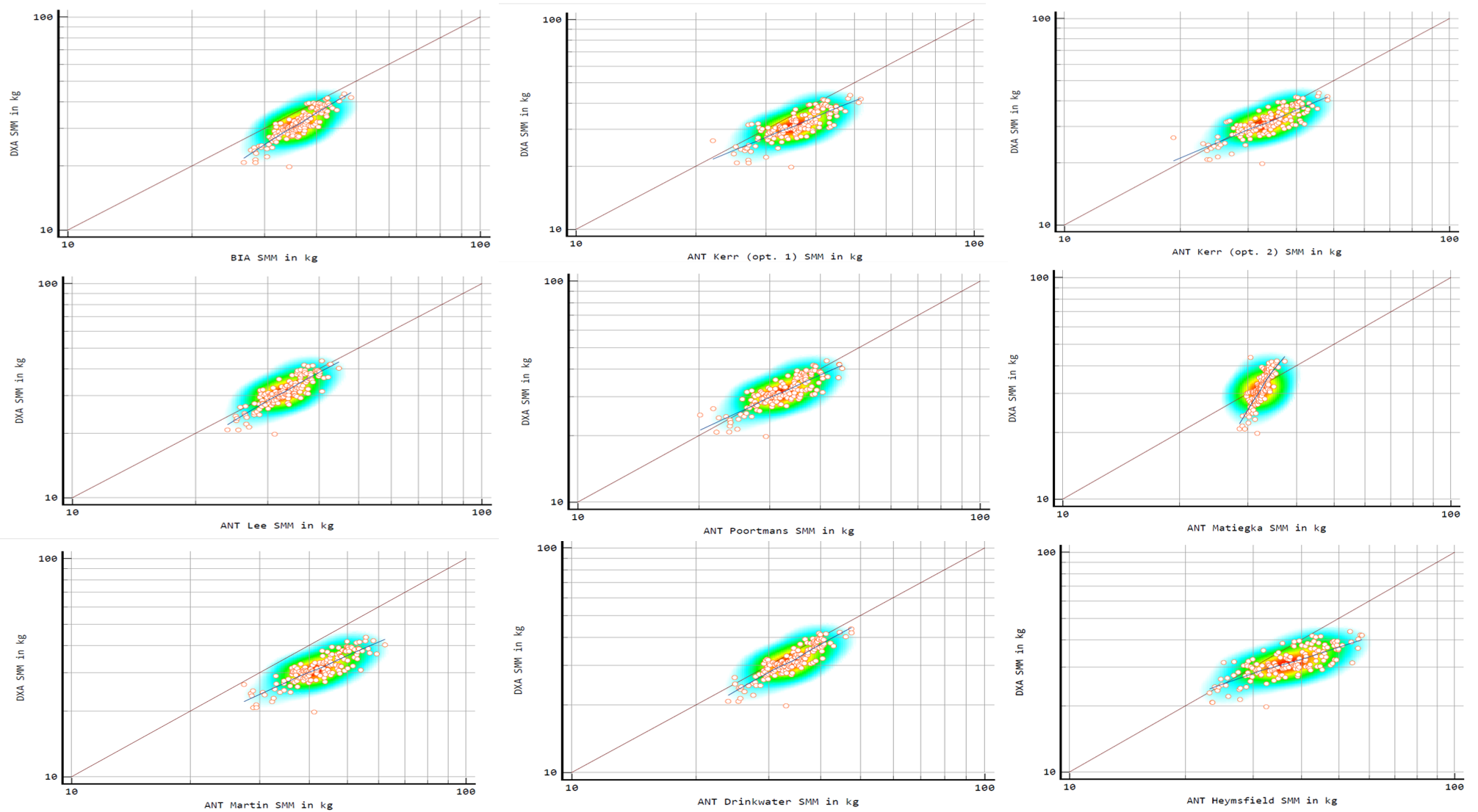

Supplement: Supplementary file 1 [file Data_Sheet_1.ZIP › Supplementary Figure 2.png]

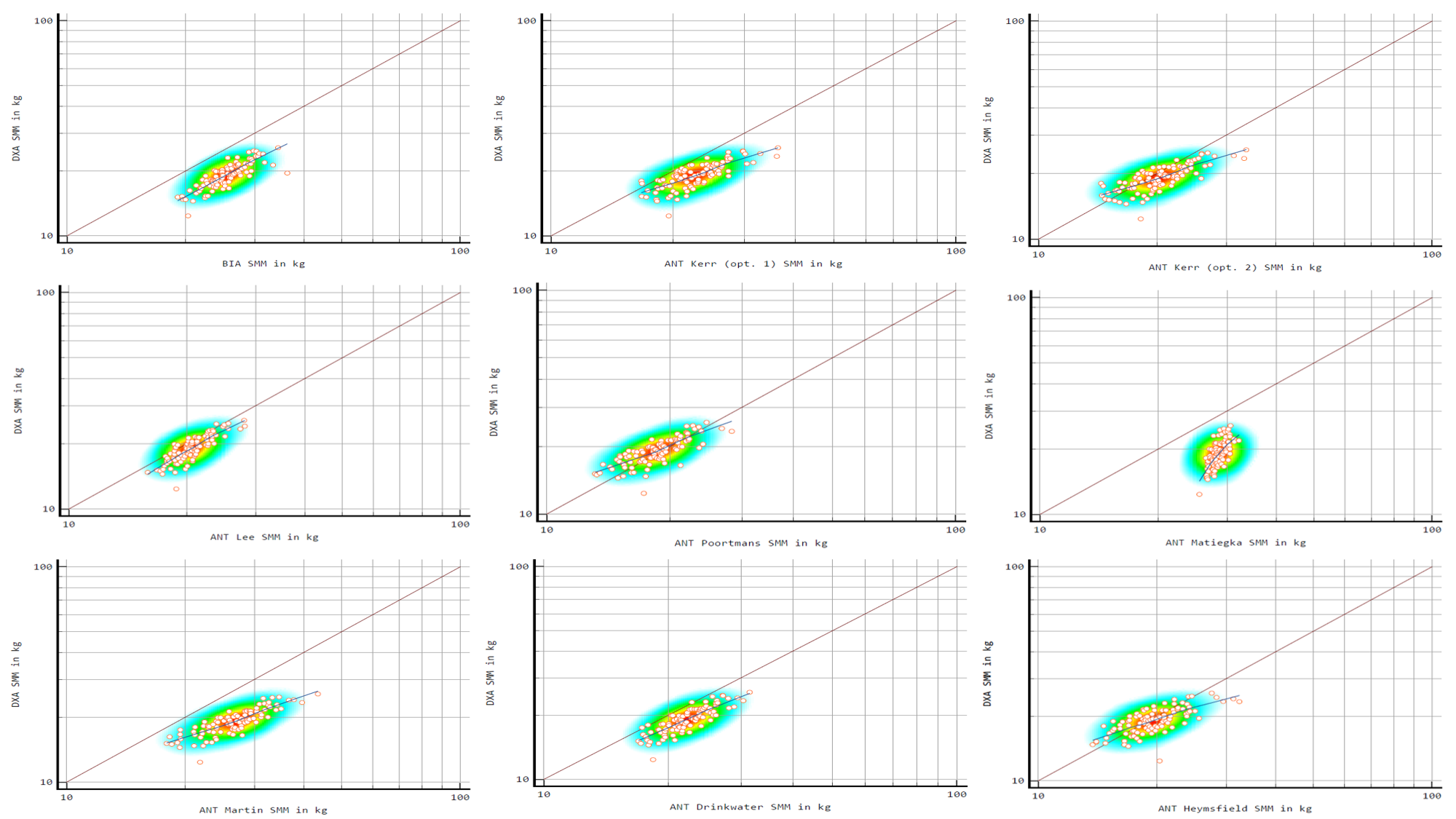

Supplement: Supplementary file 1 [file Data_Sheet_1.ZIP › Supplementary Figure 3.png]

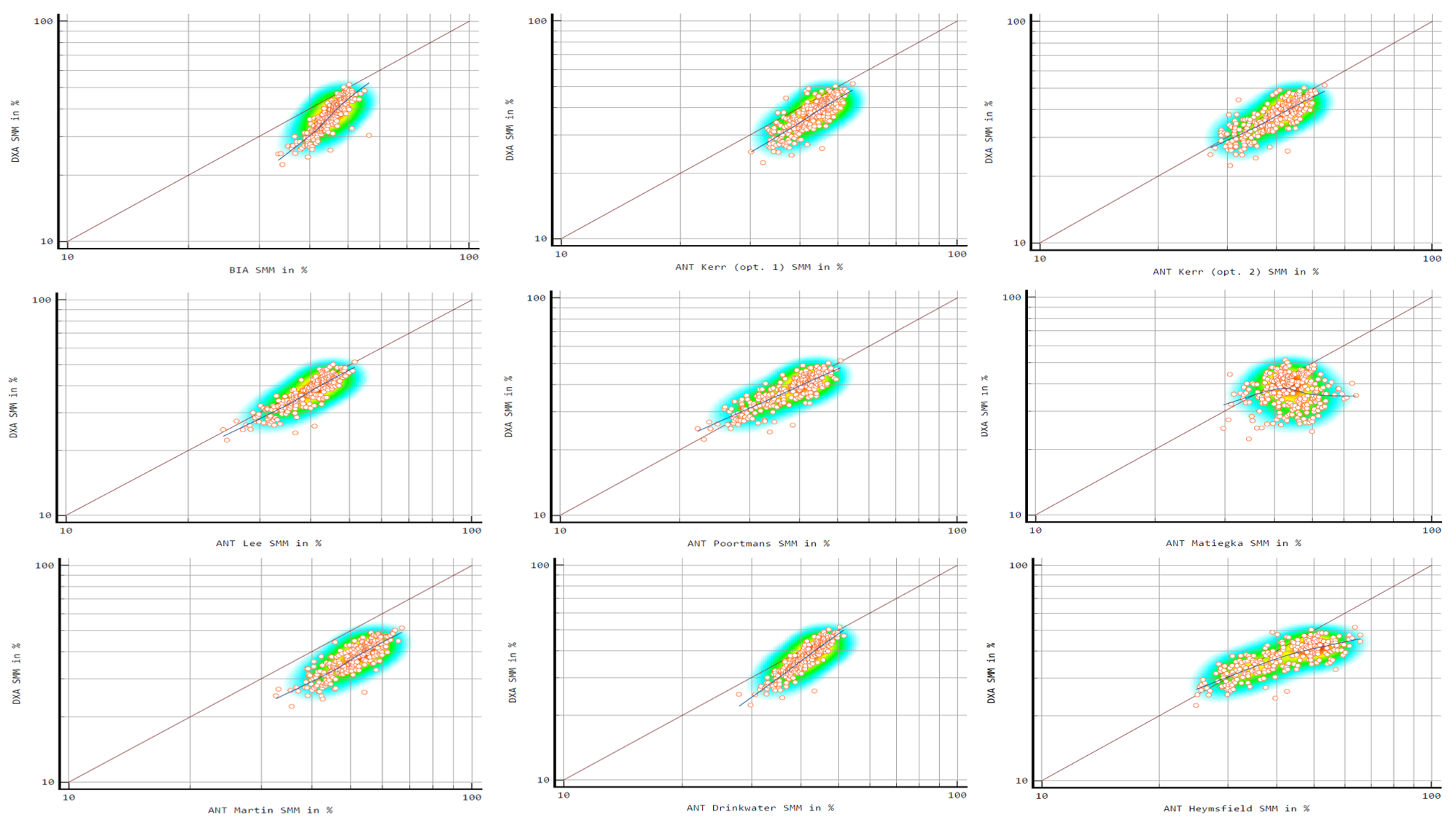

Supplement: Supplementary file 1 [file Data_Sheet_1.ZIP › Supplementary Figure 4.png]

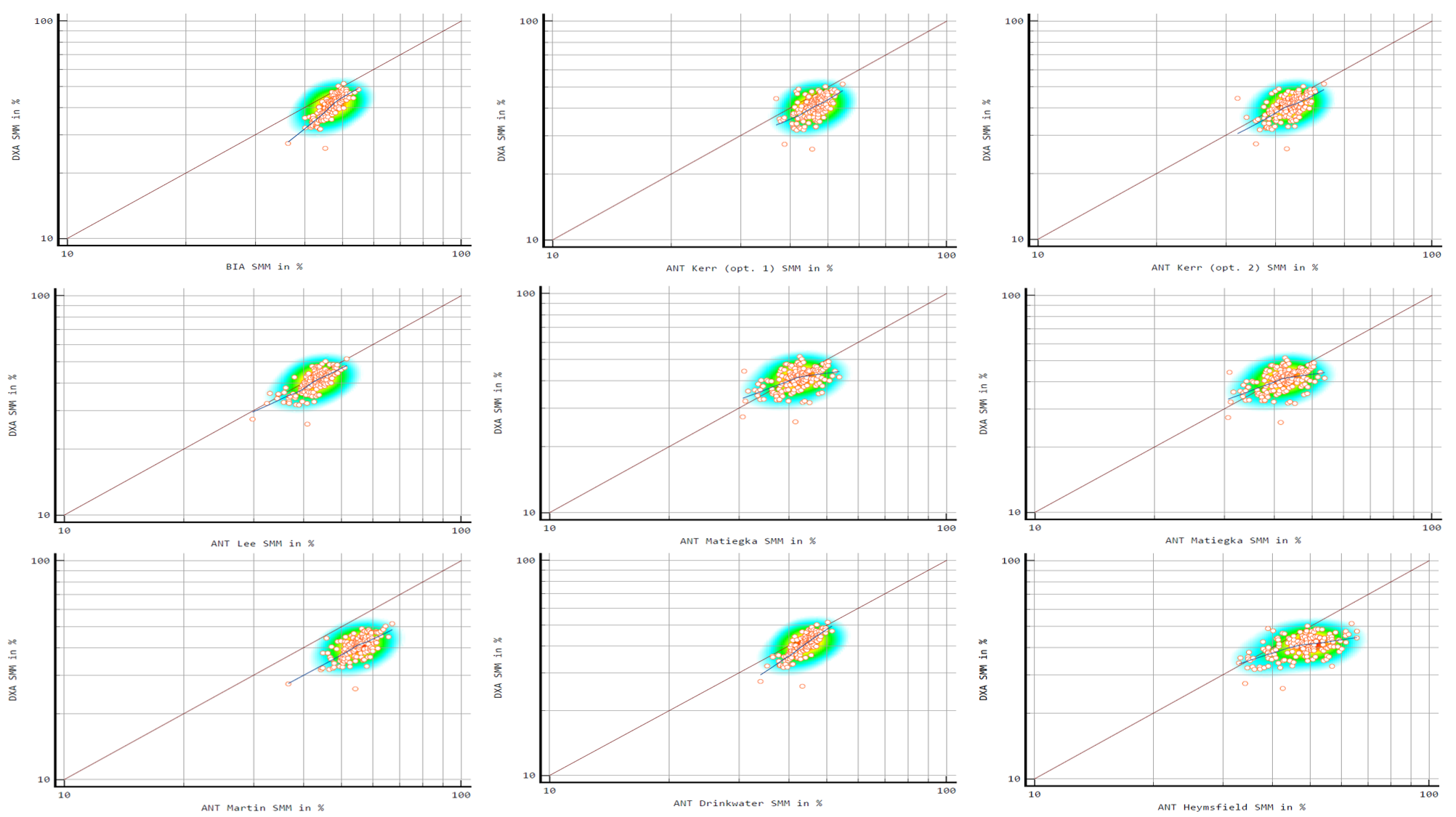

Supplement: Supplementary file 1 [file Data_Sheet_1.ZIP › Supplementary Figure 5.png]

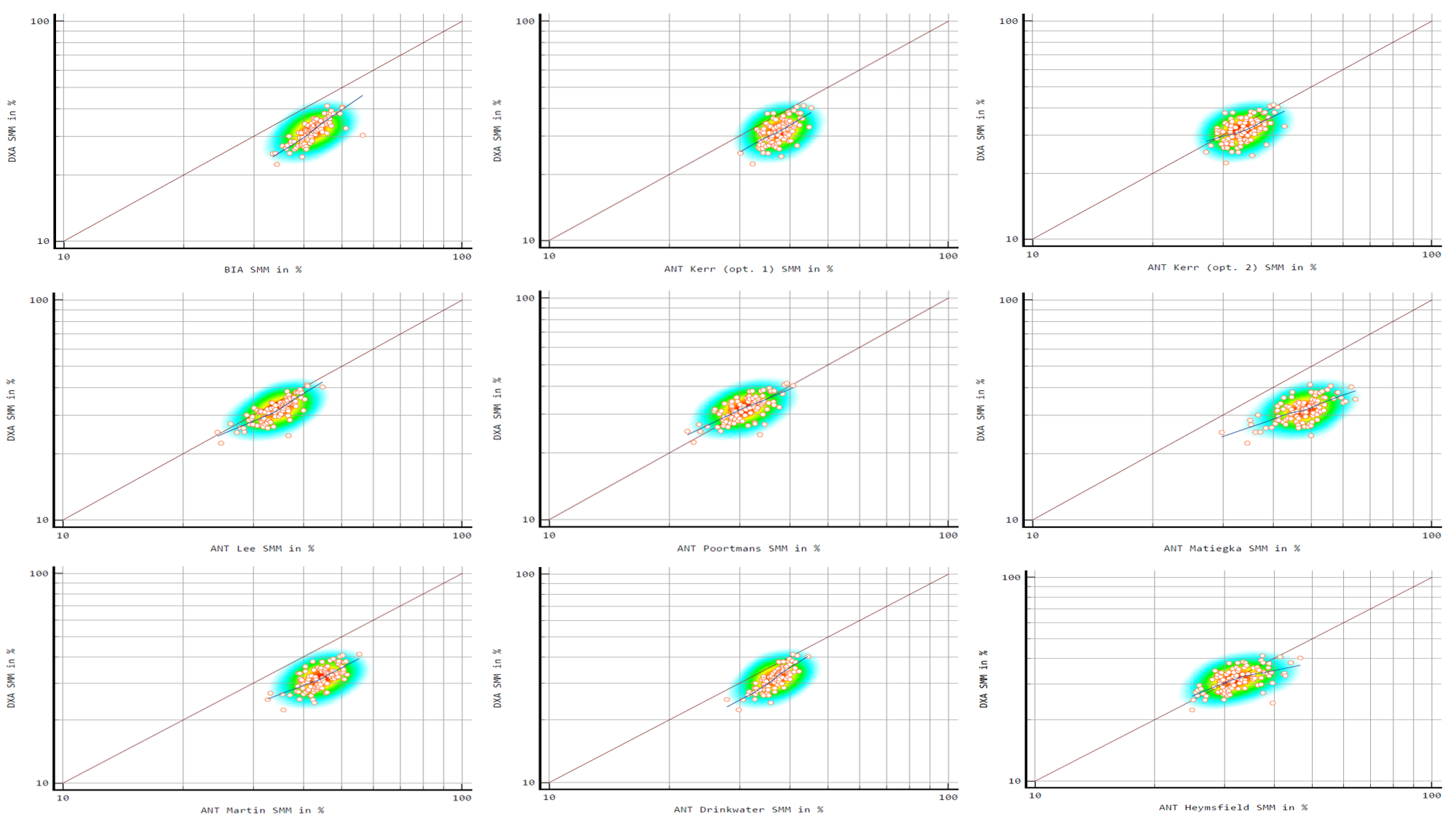

Supplement: Supplementary file 1 [file Data_Sheet_1.ZIP › Supplementary Figure 6.png]

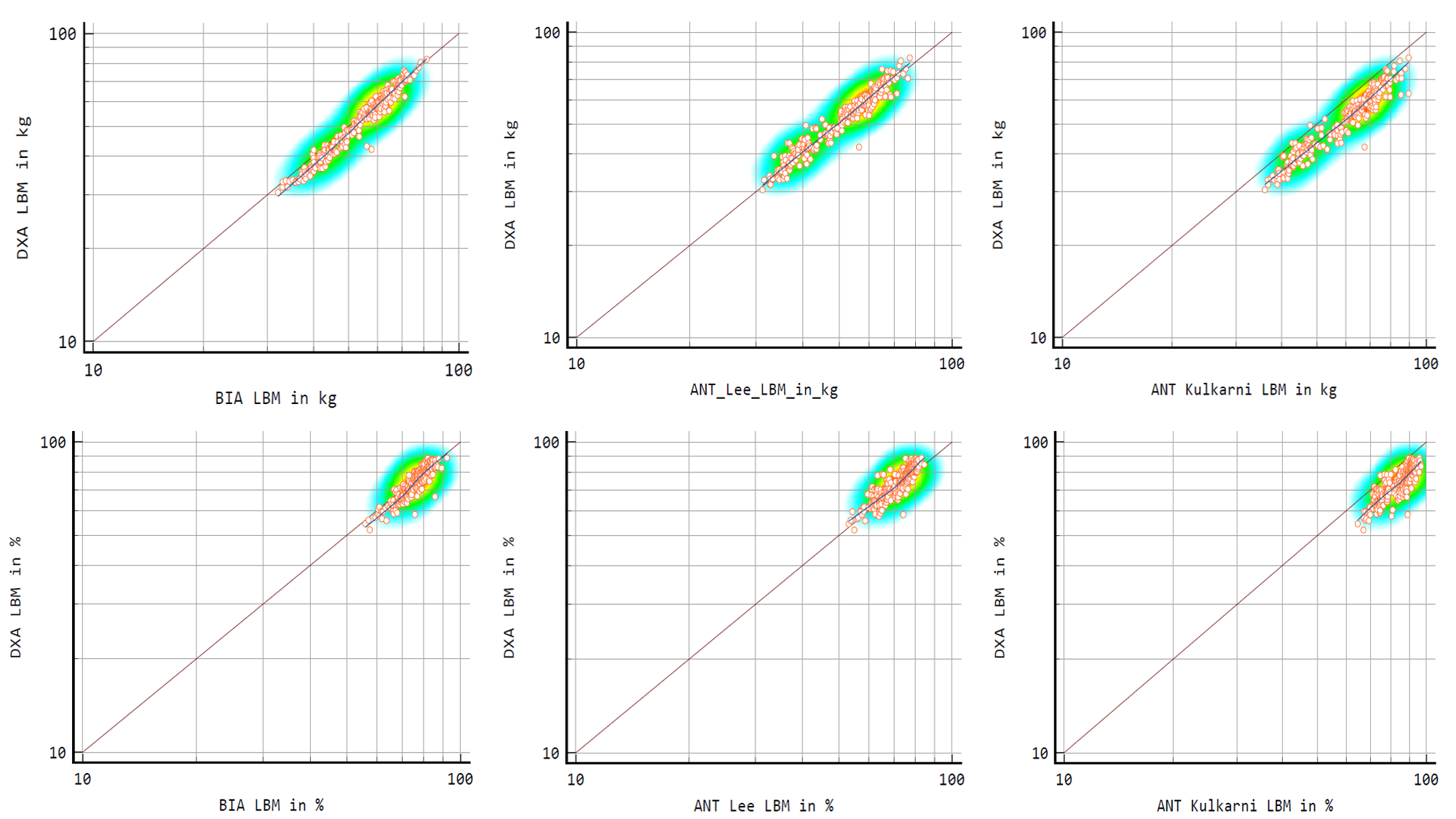

Supplement: Supplementary file 1 [file Data_Sheet_1.ZIP › Supplementary Figure 7.png]

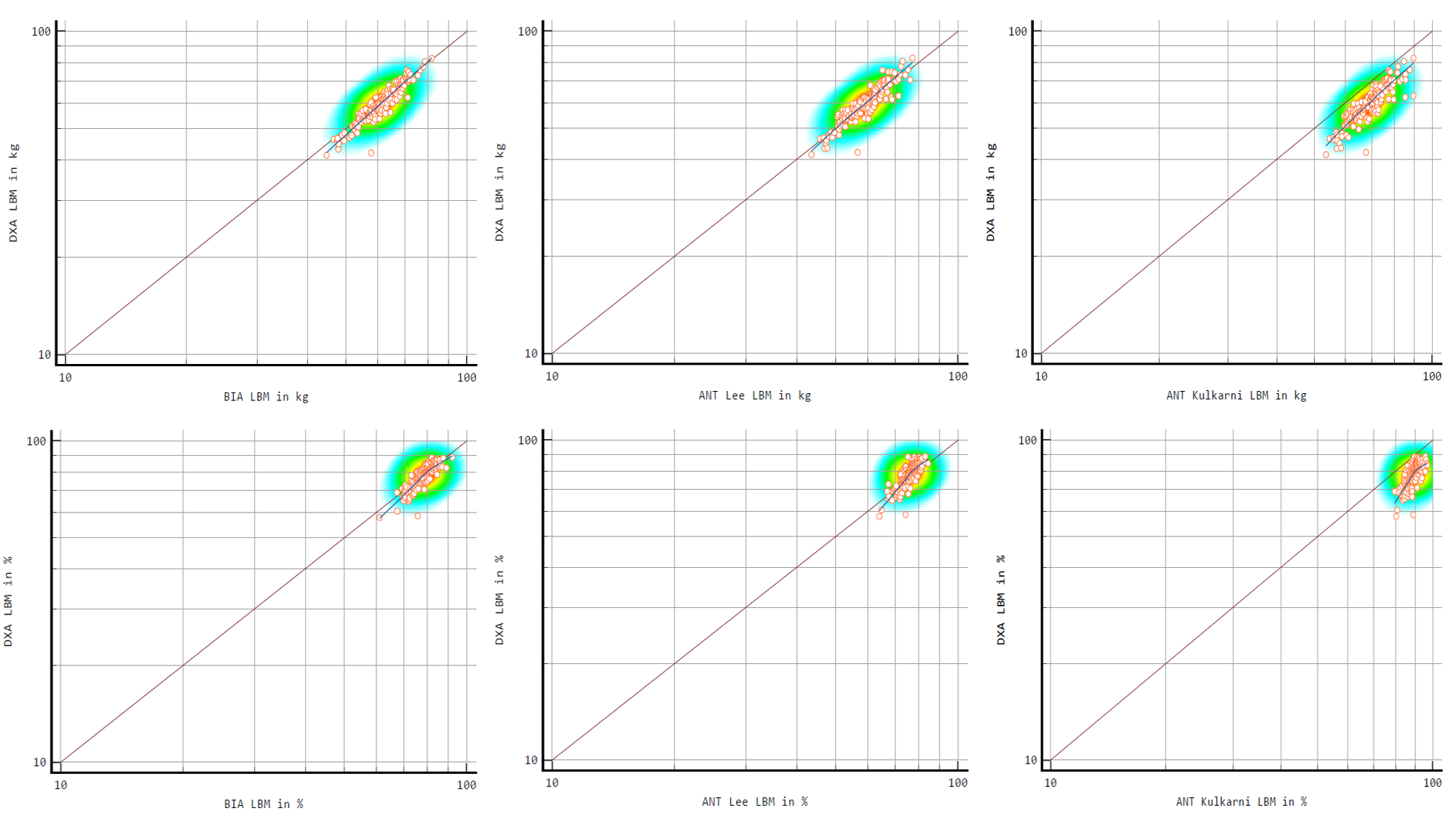

Supplement: Supplementary file 1 [file Data_Sheet_1.ZIP › Supplementary Figure 8.png]

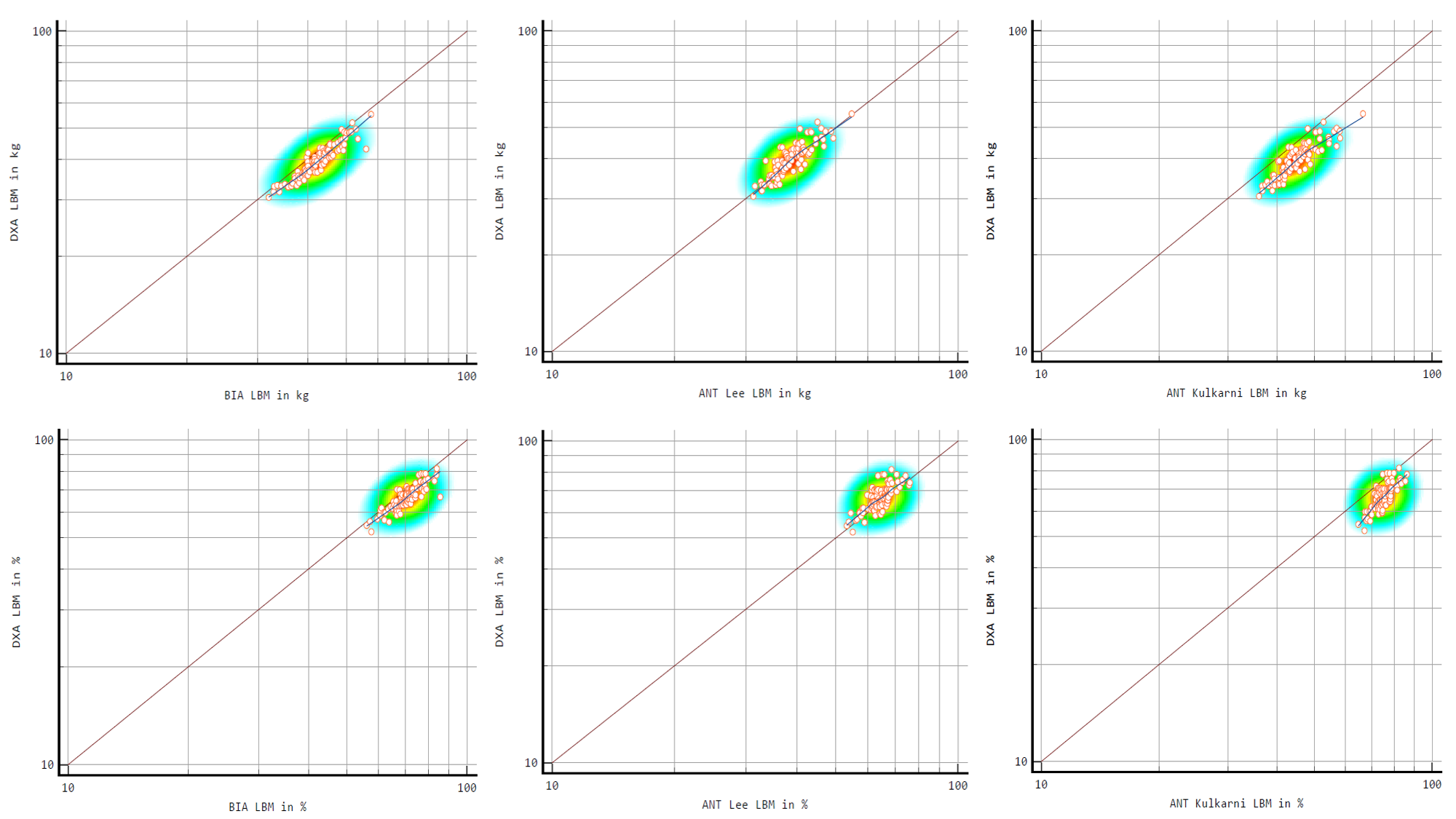

Supplement: Supplementary file 1 [file Data_Sheet_1.ZIP › Supplementary Figure 9.png]

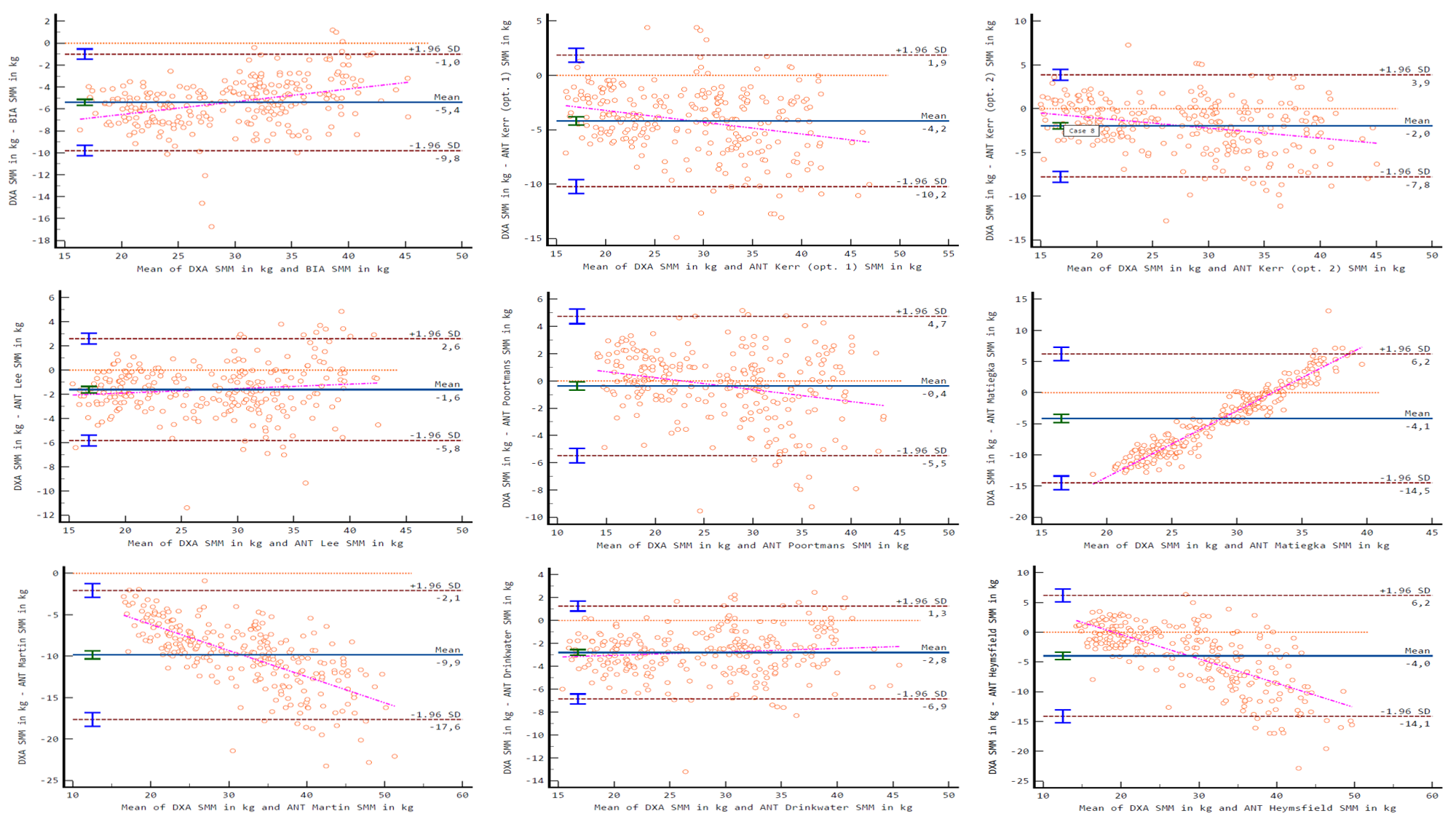

Supplement: Supplementary file 1 [file Data_Sheet_1.ZIP › Supplementary Figure 10.png]

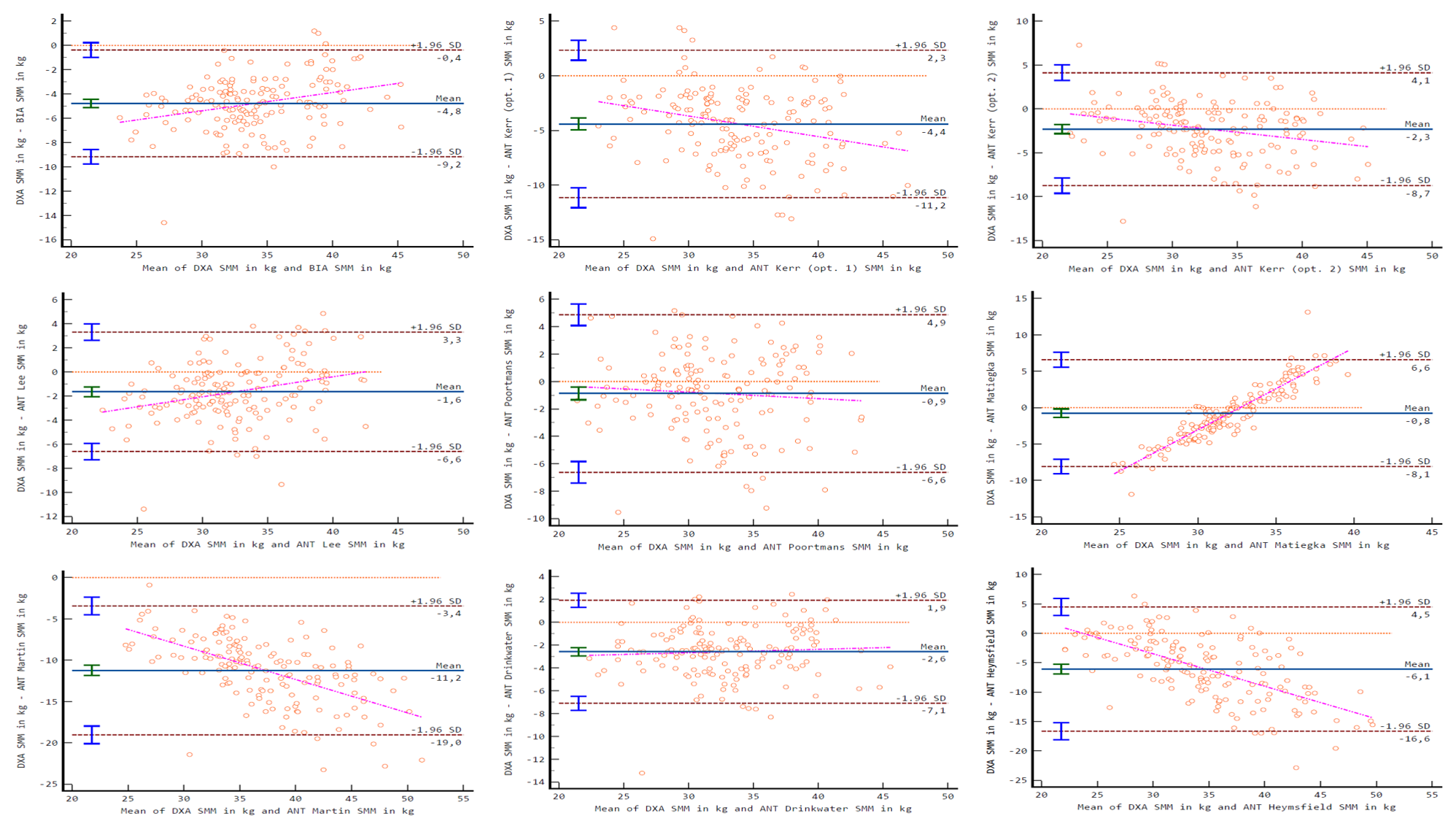

Supplement: Supplementary file 1 [file Data_Sheet_1.ZIP › Supplementary Figure 11.png]

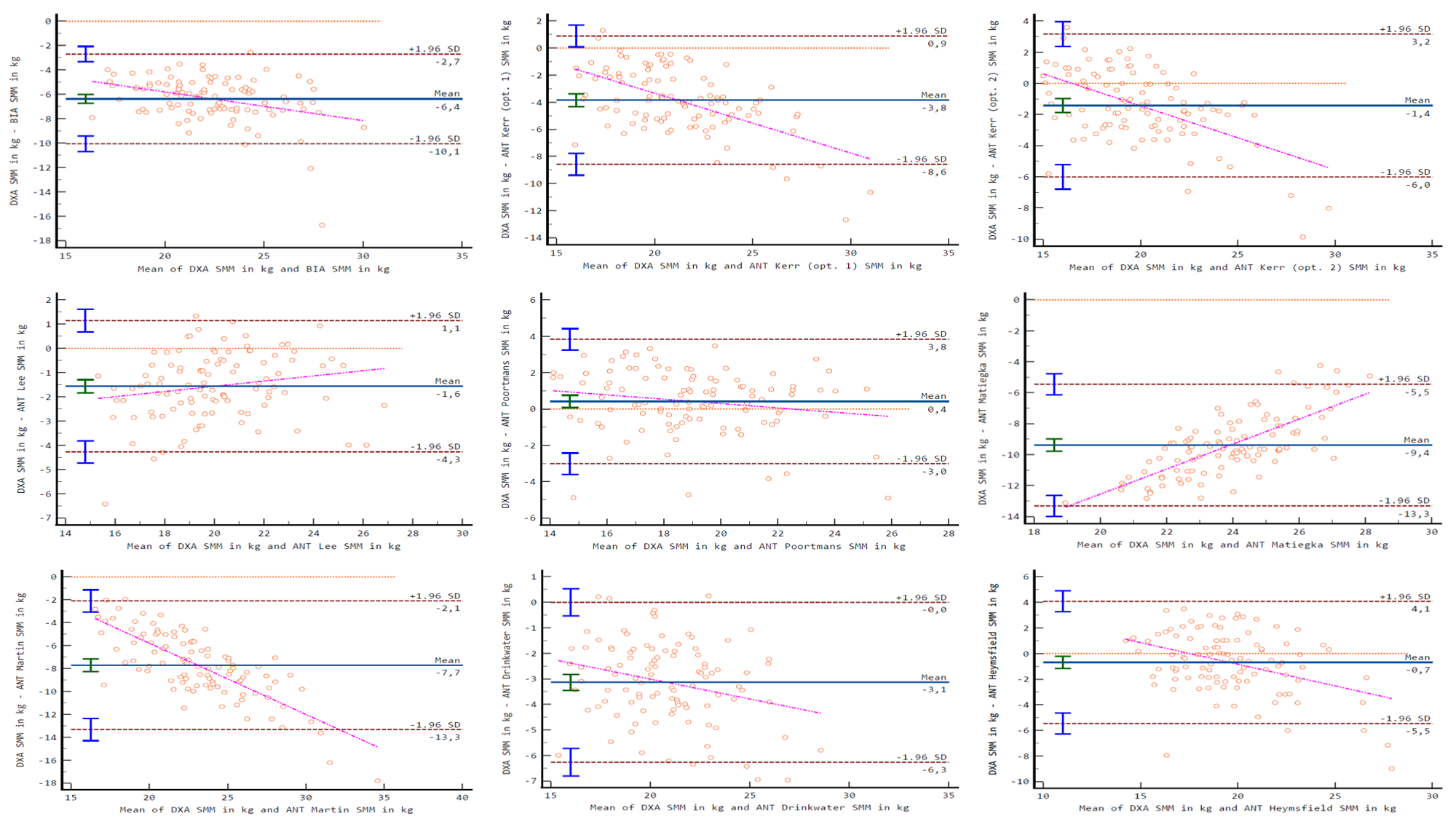

Supplement: Supplementary file 1 [file Data_Sheet_1.ZIP › Supplementary Figure 12.png]

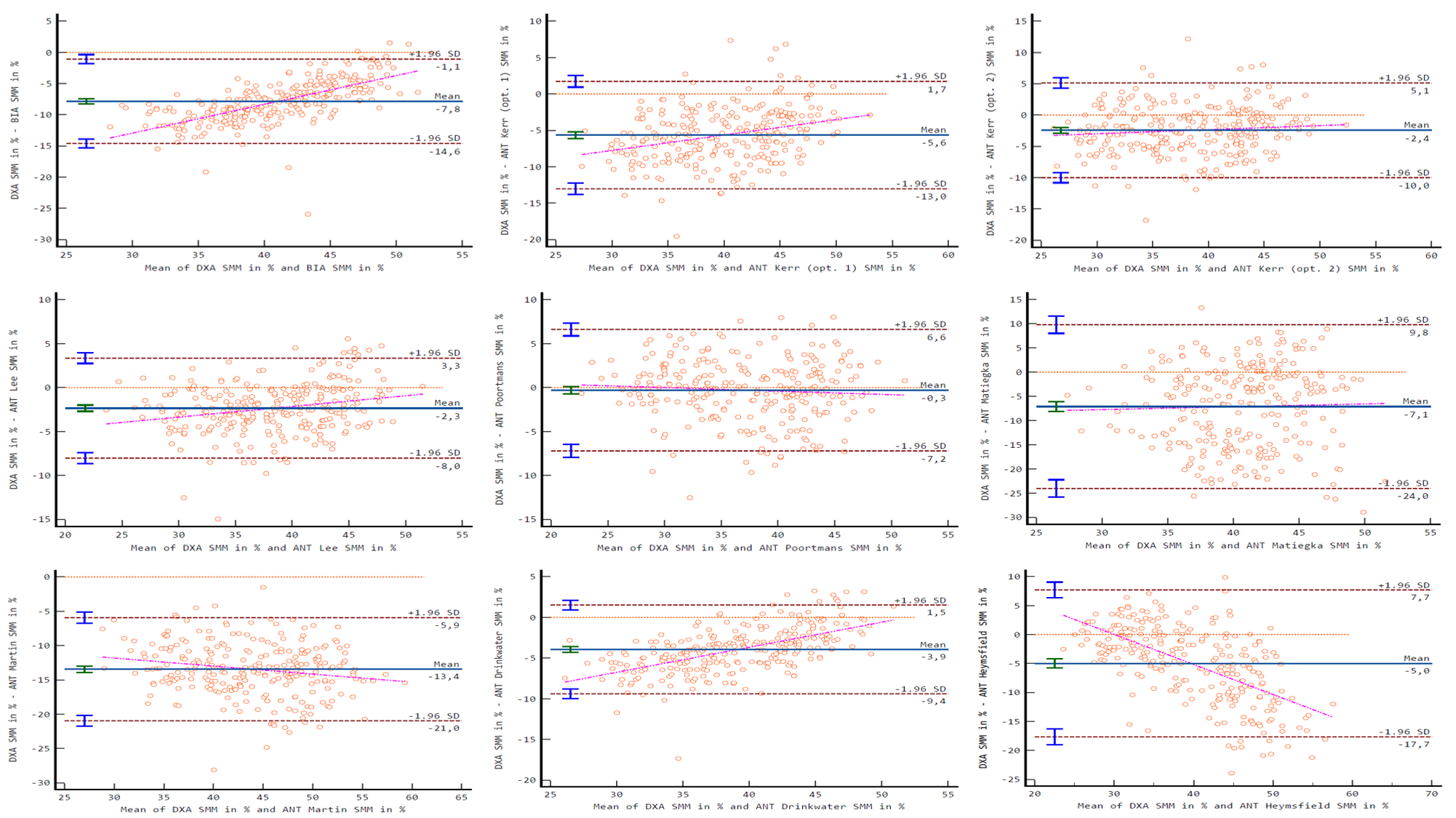

Supplement: Supplementary file 1 [file Data_Sheet_1.ZIP › Supplementary Figure 13.png]

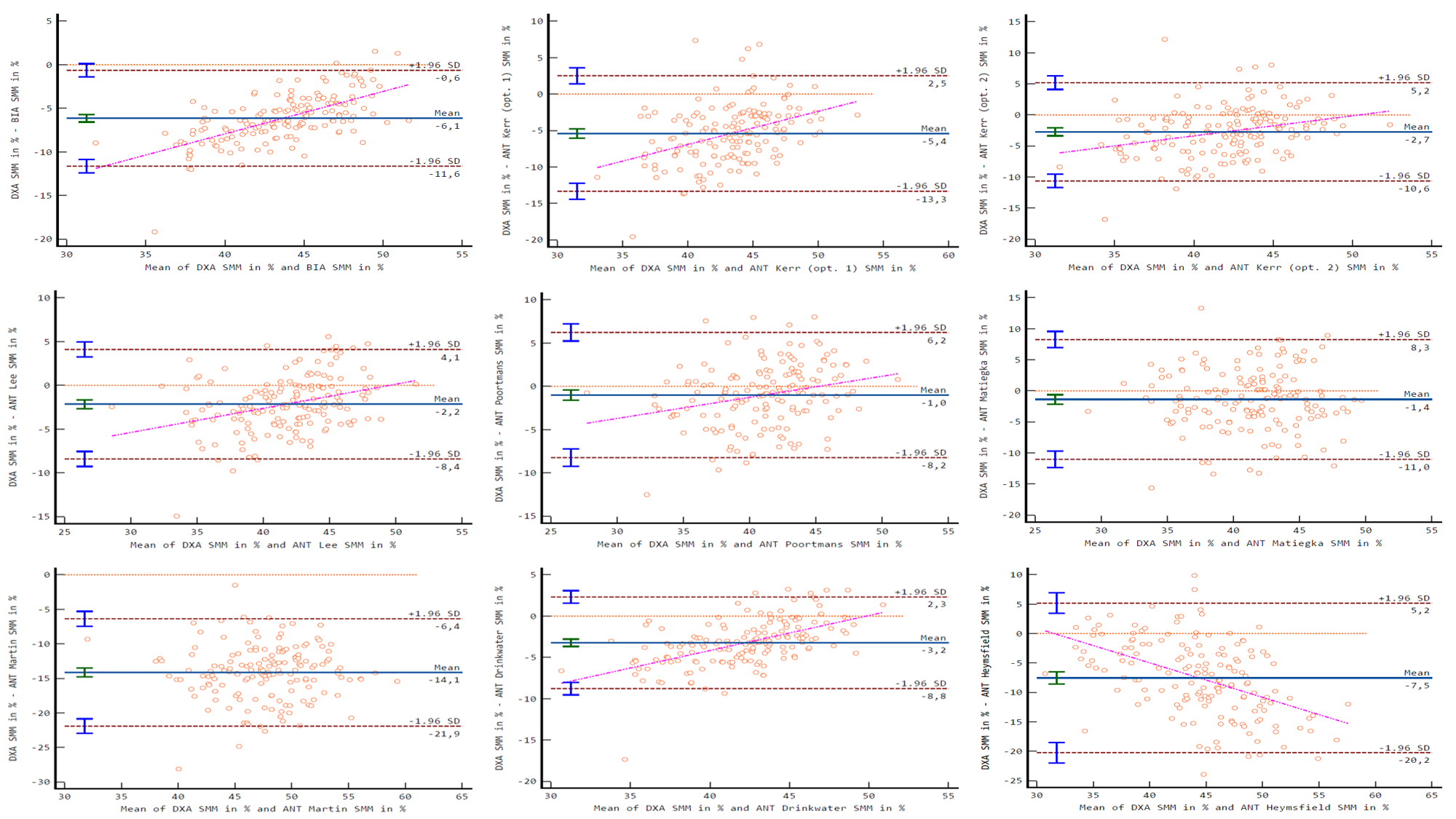

Supplement: Supplementary file 1 [file Data_Sheet_1.ZIP › Supplementary Figure 14.png]

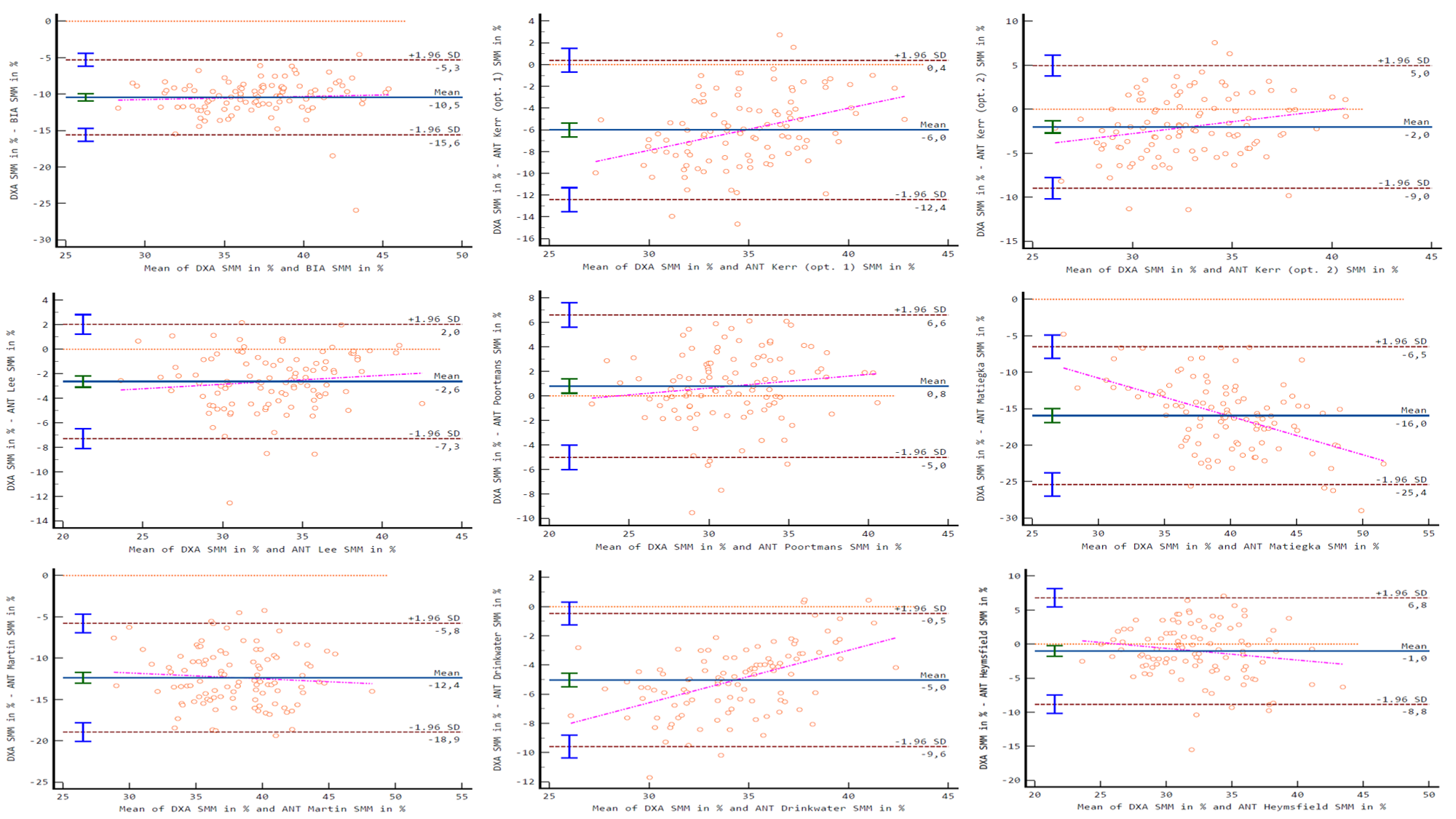

Supplement: Supplementary file 1 [file Data_Sheet_1.ZIP › Supplementary Figure 15.png]

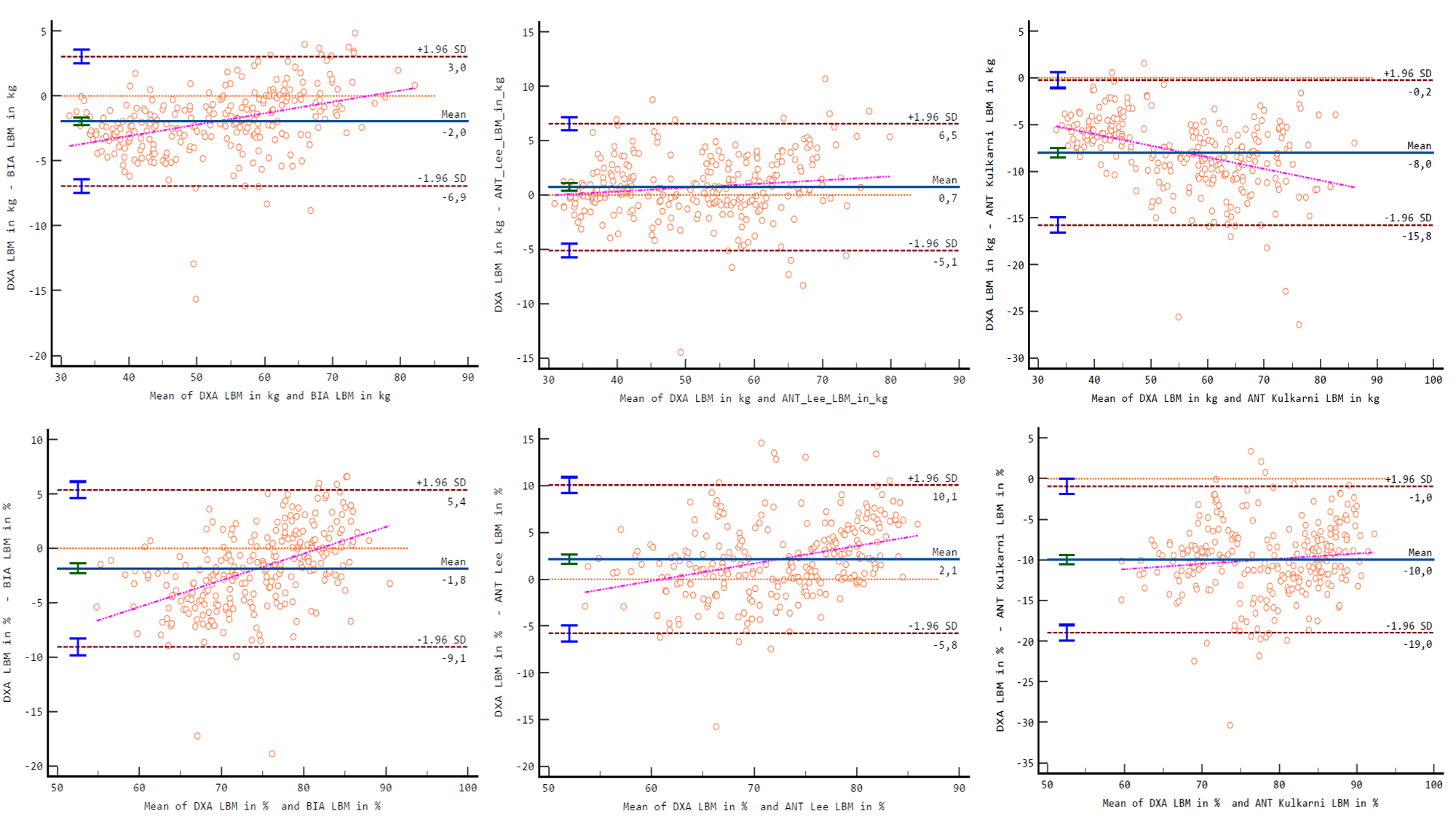

Supplement: Supplementary file 1 [file Data_Sheet_1.ZIP › Supplementary Figure 16.png]

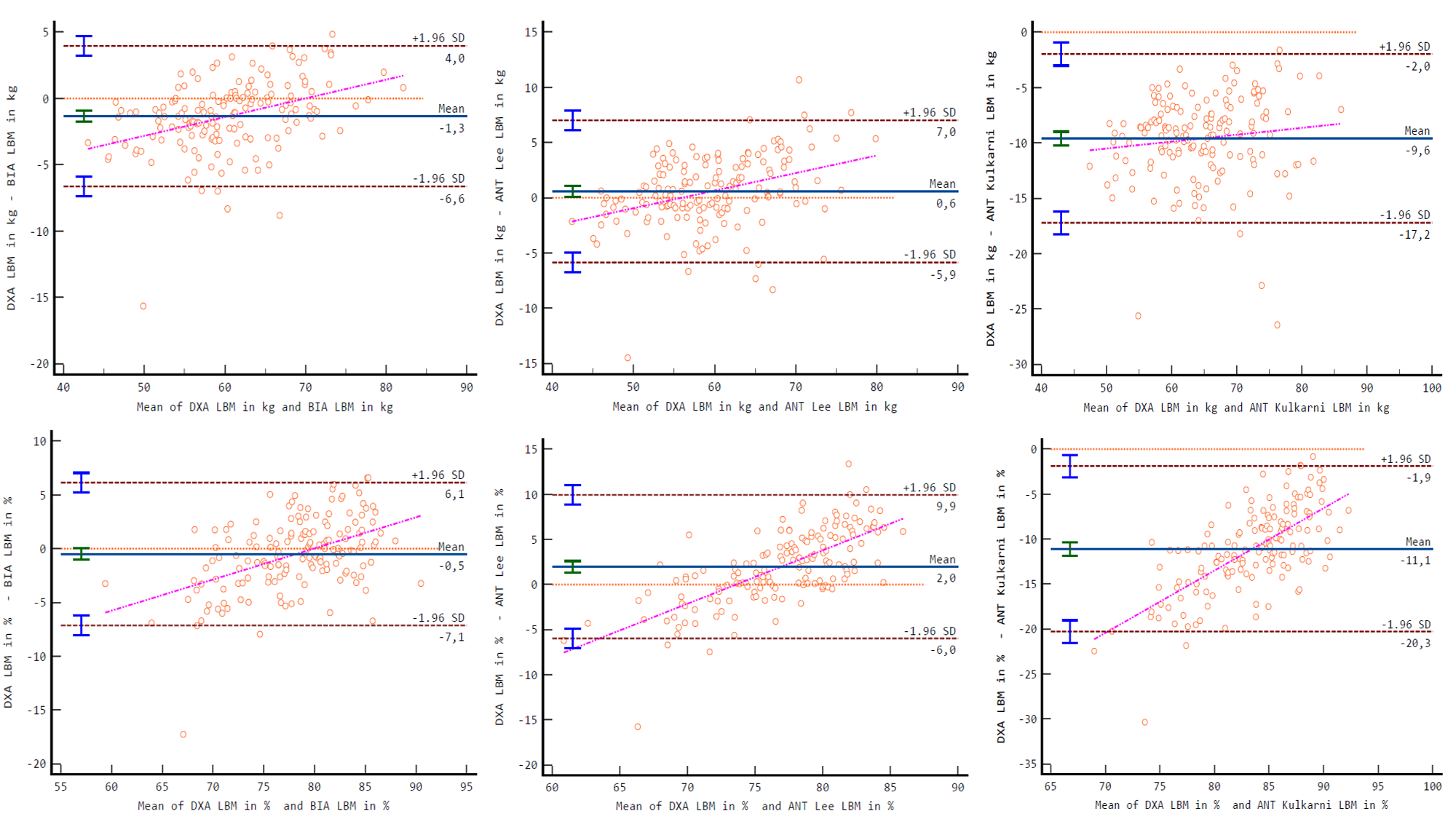

Supplement: Supplementary file 1 [file Data_Sheet_1.ZIP › Supplementary Figure 17.png]

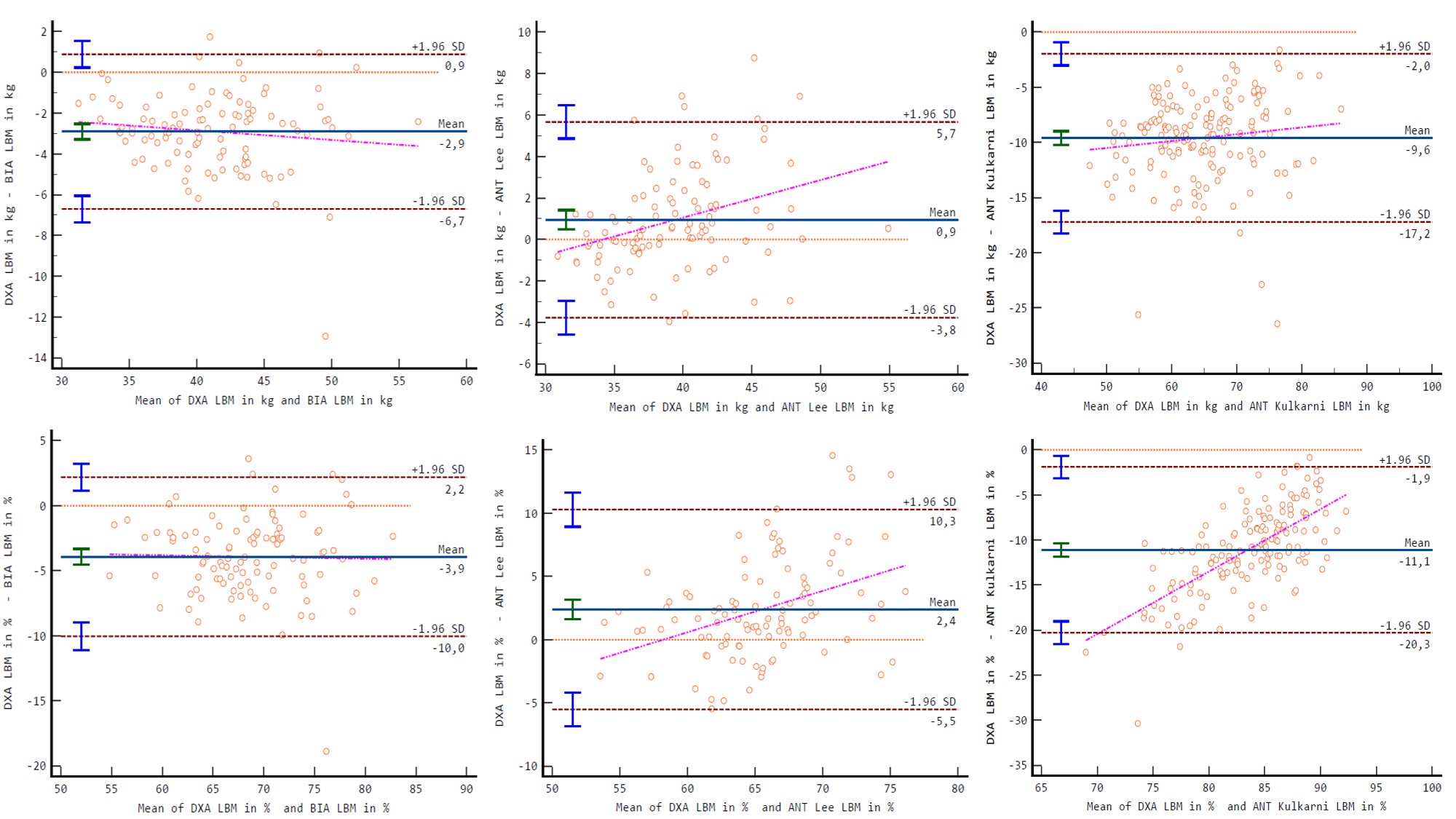

Supplement: Supplementary file 1 [file Data_Sheet_1.ZIP › Supplementary Figure 18.png]
